# Quantitative Characterization and Prediction of the Binding Determinants and Immune Escape Hotspots for Groups of Broadly Neutralizing Antibodies Against Omicron Variants: Atomistic Modeling of the SARS-CoV-2 Spike Complexes with Antibodies

**DOI:** 10.3390/biom15020249

**Published:** 2025-02-08

**Authors:** Mohammed Alshahrani, Vedant Parikh, Brandon Foley, Nishank Raisinghani, Gennady Verkhivker

**Affiliations:** 1 Keck Center for Science and Engineering, Graduate Program in Computational and Data Sciences, Schmid College of Science and Technology, Chapman University, Orange, CA 92866, USA; alshahrani@chapman.edu (M.A.); vedpar31@gmail.com (V.P.); bfoley@chapaman.edu (B.F.); nishankr@stanford.edu (N.R.); 2 Department of Structural Biology, Stanford University, Stanford, CA 94305, USA; 3 Department of Biomedical and Pharmaceutical Sciences, Chapman University School of Pharmacy, Irvine, CA 92618, USA

**Keywords:** SARS-CoV-2 spike protein, Omicron variants, antibody binding, immune escape, molecular dynamics, protein stability, mutational scanning, binding energetics, evolutionary mechanisms

## Abstract

A growing body of experimental and computational studies suggests that the cross-neutralization antibody activity against Omicron variants may be driven by the balance and tradeoff between multiple energetic factors and interaction contributions of the evolving escape hotspots involved in antigenic drift and convergent evolution. However, the dynamic and energetic details quantifying the balance and contribution of these factors, particularly the balancing nature of specific interactions formed by antibodies with epitope residues, remain largely uncharacterized. In this study, we performed molecular dynamics simulations, an ensemble-based deep mutational scanning of SARS-CoV-2 spike residues, and binding free energy computations for two distinct groups of broadly neutralizing antibodies: the E1 group (BD55-3152, BD55-3546, and BD5-5840) and the F3 group (BD55-3372, BD55-4637, and BD55-5514). Using these approaches, we examined the energetic determinants by which broadly potent antibodies can largely evade immune resistance. Our analysis revealed the emergence of a small number of immune escape positions for E1 group antibodies that correspond to the R346 and K444 positions in which the strong van der Waals and interactions act synchronously, leading to the large binding contribution. According to our results, the E1 and F3 groups of Abs effectively exploit binding hotspot clusters of hydrophobic sites that are critical for spike functions along with the selective complementary targeting of positively charged sites that are important for ACE2 binding. Together with targeting conserved epitopes, these groups of antibodies can lead expand the breadth and resilience of neutralization to the antigenic shifts associated with viral evolution. The results of this study and the energetic analysis demonstrate excellent qualitative agreement between the predicted binding hotspots and critical mutations with respect to the latest experiments on average antibody escape scores. We argue that the E1 and F3 groups of antibodies targeting binding epitopes may leverage strong hydrophobic interactions with the binding epitope hotspots that are critical for the spike stability and ACE2 binding, while escape mutations tend to emerge in sites associated with synergistically strong hydrophobic and electrostatic interactions.

## 1. Introduction

The SARS-CoV-2 spike (S) glycoprotein is a critical component of the virus, playing a central role in host cell entry, immune evasion, and viral transmission. Extensive structural and biochemical studies have provided profound insights into the mechanisms by which the S protein facilitates these processes [1,2,3,4,5,6,7,8,9]. Its remarkable conformational flexibility, particularly within the S1 subunit, enables the virus to adapt to various stages of the viral entry process while evading immune surveillance. This dynamic nature contributes to the high transmissibility and pathogenicity of SARS-CoV-2, making it a key target for therapeutic and vaccine development. The S glycoprotein is composed of two subunits: S1 and S2. The S1 subunit, which is responsible for receptor binding, contains several critical domains. The N-Terminal Domain (NTD) is involved in initial host cell attachment and plays a role in stabilizing the S protein and modulating its conformational dynamics. The Receptor-Binding Domain (RBD) is the primary region responsible for binding to the host cell receptor, angiotensin-converting enzyme 2 (ACE2). Its ability to transition between “closed” and “open” states is crucial for viral entry. Subdomains SD1 and SD2 are structurally conserved regions that stabilize the prefusion conformation of the S protein and facilitate the transition to the post-fusion state, which is necessary for membrane fusion and viral entry [10,11,12,13,14,15]. The conformational flexibility of the S1 subunit, particularly within the NTD and RBD, allows the virus to effectively engage with host cell receptors while evading immune surveillance. This structural variability is a key factor in the virus’s ability to adapt to selective pressures and maintain high transmissibility. The S protein undergoes dynamic transitions between closed and open states, which are driven by conformational changes in the NTD and RBD. These transitions regulate the accessibility of the RBD, enabling the virus to balance efficient ACE2 binding with immune evasion. Mutations within the S1 subunit, particularly in the RBD, can induce structural alterations that affect the protein’s stability, conformational dynamics, and immune evasion capabilities. Mutations in the RBD can enhance the virus’s ability to evade neutralizing antibodies (Abs) by altering the distribution of functional states and reducing the binding affinity of Abs. The structural variability introduced by these mutations can make it more challenging for the host immune system to mount an effective response.

Biophysical studies have shown that thermodynamic and kinetic factors govern these conformational changes, highlighting the importance of understanding the structural basis of S protein dynamics for developing effective therapeutics and vaccines [16,17,18]. Advances in cryo-electron microscopy (cryo-EM) and X-ray crystallography have provided high-resolution images of the S protein’s structure in various functional states, including its interactions with antibodies [19,20,21,22,23,24,25]. These studies have revealed how variants of concern (VOCs) induce structural changes in the dynamic equilibrium of the S protein, influencing its functional states and binding affinities with different classes of Abs. Structural alterations in VOCs can shift the distribution of functional states, affecting the accessibility of the RBD and the efficacy of neutralizing antibodies. These insights have been instrumental in understanding how mutations in the S protein contribute to immune evasion and increased transmissibility. The evolution of SARS-CoV-2, particularly within the Omicron lineage, has been characterized by the emergence of subvariants with enhanced immune evasion and transmissibility. The XBB.1.5 subvariant emerged through recombination events within the BA.2 lineage, leading to enhanced growth and increased transmissibility [26,27,28]. This is attributed to its improved binding affinity for ACE2 and resistance to neutralization by antibodies. The “FLip” variants are characterized by the convergent acquisition of the L455F and F456L double mutations [29,30,31,32,33]. These mutations provide a significant growth advantage, underscoring the evolutionary pressure to optimize immune evasion while maintaining ACE2 binding [33]. The BA.2.86 subvariant, derived from BA.2, exhibits substantial genetic divergence from earlier Omicron variants and demonstrates enhanced evasion against RBD-targeted antibodies compared to XBB.1.5 and EG.5.1, highlighting the ongoing evolution of immune escape mechanisms [34,35,36,37,38].

The current study is focused primarily on the JN.1 variant of BA.2.86, which emerged independently of Omicron BA.2 and carries an additional L455S mutation that enhances its ability to evade the immune system [39,40]. A comparative biochemical analysis using surface plasmon resonance (SPR) assays revealed a significant reduction in ACE2’s binding affinity to JN.1 and increased evasion against RBD class-1 Abs such as S2K146 and Omi-18, as well as the class-3 Abs S309 [34,39]. A series of SARS-CoV-2 variants with mutations at the L455, F456, and R346 positions include the “SLip” variant, which carries L455S along with an additional F456L mutation. More recently, the “FLiRT” variant has appeared, featuring an additional R346T mutation on the SLip backbone. Studies have shown that the SLip and FLiRT subvariants of JN.1 can enhance the escape of JN.1-derived variants from neutralizing Abs [40,41,42,43]. JN.1 has evolved into multiple subvariants, each with distinct mutations that contribute to immune evasion and transmissibility: KP.2 carries mutations R346T, F456L, and V1104L; KP.3 (FLuQE) features mutations R346T, L455S, F456L, Q493E, and V1104L, showing strong growth and immune evasion [44]; LB.1 (JN.1 + S:S31-, S:Q183H, S:R346T, S:F456L) and KP.2.3 subvariants (JN.1+ S:R346T, S:H146Q, S:S31) contribute to the increased immune evasion and the increased relative effective reproduction number (Rt), which is a measure of the transmission potential of a virus, indicating the average number of new infections caused by a single infected individual at a specific time [45]. Among these, the KP.2 and KP.3 variants share the F456L mutation and KP.3 has emerged as the most immune-evasive and fastest-growing JN.1 sublineage, largely due to the F456L mutation, which plays a critical role in the antibody (Ab) escape [46]. Recent cryo-EM studies of JN.1, KP.2, and KP.3 have revealed the epistatic effects of mutations Q493E and F456L on ACE2 binding [47,48]. Epistatic effects refer to the interaction between mutations, leading to their non-additive contribution, where the effect of one mutation depends on the presence of another mutation. Notably, the F456L mutation enhances the binding potential of Q493E, leading to stronger receptor interactions and providing an evolutionary advantage for the incorporation of additional immune-evasive mutation [47,48]. A newly emerged recombinant variant, XEC, derived from KS.1.1/KP.3.3, carries additional mutations (F59S and T22N) in the NTD [49,50]. Functional assays indicate that XEC has higher infectivity and more robust immune resistance compared to KP.3, positioning it as a potential candidate to become the next dominant strain [51,52].

High-throughput yeast display screening and deep mutational scanning (DMS) have been used to map the escape mutation profiles of the RBD residues for human anti-RBD neutralizing Abs, allowing for the functionally significant classification of neutralizing Abs into six epitope groups (A–F) [53]. The recent seminal study exploited this approach to characterize the epitope distribution of Abs elicited by post-vaccination BA.1 infection and identified the mutational escape profiles for 1640 RBD-binding Abs, which were classified into 12 epitope groups [54]. In this classification, groups A–C consist of Abs targeting the ACE2-binding motif. Group D Abs, such as REGN-10987, LY-CoV1404, and COV2-2130, bind to the epitope 440–449 on the RBD and are further divided into D1 and D2 subgroups. Groups E and F are subdivided into E1–E3 and F1–F3, respectively, covering the front and back of the RBD. These groups correspond to class 3 and class 4 Abs according to the earlier classification [55]. Group E Abs are sensitive to mutations of G339, T345, and R346, while neutralization by group F Abs can be compromised by changes in F374, T376, and K378 and, in some Abs in this group, to V503 and G504, similar to the epitopes of S2X259, suggesting that they can compete with ACE2 [53,54].

The molecular mechanisms underlying the broadly neutralizing Abs induced by XBB/JN.1 infections were determined via high-throughput yeast-display-based DMS assays and the escape mutation profiles of a total of 2688 Abs, including 1874 isolated from XBB/JN.1 infection cohorts, were determined, producing a total of 22 Ab clusters [56]. This study showed that JN.1 reinfections elicit Abs that offer better protection against emerging variants due to the enrichment of class 1 Abs and the potential of F3 Omicron-specific Abs [56]. A seminal study by Cao and colleagues identified four E1 group Abs, BD55-3546, BD55-3152, BD55-5585, BD55-5549 and BD55-5840 (SA58) Abs, as well as F3 Abs BD55-4637, BD55-3372, BD55-5483, and BD55-5514 (SA55) [57]. This study revealed the non-competing RBD binding of SA55 and SA58 using SPR competition assays and showed that while the accumulation of mutations on R346 and K444 may affect the neutralization of SA58, SA55 could efficiently neutralize all escaping mutants, including convergent mutations, on the RBD of BQ.1, BQ.1.1, and XBB [57]. Together, the results from recent studies revealed the evolving Ab response to Omicron’s antigenic shift from XBB to JN.1, where groups F3, A1, B, and D3 Abs retain their potency and neutralizing activity against JN.1 subvariants, whereas A2, D2, D4, and E1/E2.1 largely escape [46,54,57]. The latest studies show that repeated Omicron infection stimulates a higher level of Omicron-specific Abs that have distinct RBD epitopes and escaping mutations compared to WT-induced monoclonal Abs [46,58].

The two recently discovered Abs, CYFN1006-1 and CYFN1006-2, demonstrated consistent neutralization of all tested SARS-CoV-2 variants, outperforming SA55 [59]. These Abs have binding epitopes overlapping with LY-CoV1404, REGN10987, and S309, located on the outer surface of the RBD. They bind to a different RBD region compared to SA55, suggesting that combining SA55 and CYFN1006-1 could be beneficial against JN.1, KP.2, KP.3, and evolving SARS-CoV-2 mutants [59]. A yeast-display system combined with a machine learning (ML)-guided approach for library design enabled an investigation of a larger number of Ab variants and the identification of a class 1 human Ab designated as VIR-7229, which targets the receptor-binding motif (RBM), potentially neutralizing SARS-CoV-2 variants, including EG.5, BA.2.86, and JN.1 [60]. The structures of VIR-7229-bound to XBB.1.5 and EG.5 structures showed that the VIR-7229 interactions can accommodate both F456 and L456 in the corresponding genetic backgrounds and tolerate an extraordinary epitope variability, exhibiting a high barrier against the emergence of resistance, which is partly attributed to their high binding affinity [60]. High-throughput DMS assays were used to analyze 1637 potent Abs against eight major SARS-CoV-2 variants, including B.1 (D614G), Omicron BA.1, BA.2, BA.5, BQ.1.1, XBB.1.5, HK.3, and JN.1 [61]. The study found that 296 Abs effectively neutralized XBB.1.5 and 147 neutralized JN.1. One notable discovery was BD55-1205, a class 1/group A1 Ab that showed broad neutralization with high affinity ranging from 1 pM to 18 nM against all major SARS-CoV-2 variants tested, including XBB-, BA.2.86-, and JN.1-derived subvariants, and a high barrier to escape [61]. Collectively, this growing number of structural, functional, and biophysical studies reveals the diversity of the mechanistic scenarios underlying Ab binding and catalogs the RBD escape mutations for a wide range of Abs, unveiling the distinct signatures of Ab-resistant mutational hotspots.

Computer simulations have revolutionized our understanding of the SARS-CoV-2 S protein, particularly its dynamics, interactions with the ACE2 receptor, and mechanisms of Ab resistance. By leveraging molecular dynamics (MD) simulations, Markov state models (MSM), and advanced computational tools like AlphaFold2, the version number is 2.3.2, researchers have gained atomic-level insights into the structural and functional properties of the S protein and its complexes. These studies have revealed critical details about the evolutionary mechanisms driving the emergence of Omicron variants, particularly in their ability to balance ACE2 binding affinity and immune evasion. Convergent evolution has played a pivotal role in shaping the evolutionary trajectory of SARS-CoV-2, particularly in the Omicron lineage. This process has led to the repeated emergence of mutations at specific residues, such as G446S, F486V, F486P, F486S, and F490S, which exhibit epistatic couplings with major stability and binding affinity hotspots [62]. MD simulations and MSM analysis have characterized the conformational landscapes of the Omicron variants and their complexes [63]. Additionally, mutational scanning and binding analyses of Omicron XBB spike variants have provided a quantitative rationale for experimental observations, highlighting the importance of epistatic interactions between nearby binding hotspots [64,65]. We combined AlphaFold2-based atomistic predictions of the structures and conformational ensembles of the S complexes with the ACE2 for the most dominant Omicron variants, JN.1, KP.1, KP.2 and KP.3, to examine the mechanisms underlying the role of convergent evolution hotspots in balancing ACE2 binding and Ab evasion [66]. This study identified binding energy hotspots and characterized the epistatic interactions between convergent mutational sites at the L455, F456, Q493 positions that can protect and restore ACE2 binding affinity while conferring a beneficial immune escape. The S protein functions as an allosteric regulatory machinery, leveraging the intrinsic plasticity of its functional regions to modulate binding and regulatory functions [67,68,69,70,71]. Computational studies combining all-atom MD simulations, ensemble-based mutational scanning, and perturbation-based network profiling have revealed that the escape mechanisms of ultrapotent antibodies are not solely determined by binding interactions but are also influenced by structural stability, binding strength, and long-range allosteric communications [72].

The convergent evolution of SARS-CoV-2, particularly within the Omicron lineage, reflects a delicate balance between immune evasion, ACE2 binding affinity, and conformational adaptability [73]. Electrostatic interactions have emerged as a critical thermodynamic force governing the binding of the S protein to ACE2 and its resistance to antibodies [74,75,76]. The accumulation of positively charged residues on the RBD of many Omicron variants reflects evolutionary adaptations that enhance ACE2 binding while promoting immune evasion. These studies have highlighted the role of electrostatic changes in immune evasion, particularly in JN.1 variants. The JN.1 variant, characterized by an accumulation of lysine residues (e.g., R403K, N460K, N481K, A484K, F486P, R493Q, and E554K), demonstrates significant electrostatic alterations that contribute to reduced ACE2 affinity and increased Ab evasion [77,78]. Many studies have suggested functionally balanced substitutions that optimize the tradeoffs between immune evasion, high ACE2 affinity, and sufficient conformational adaptability might be a common strategy of the virus evolution and serve as a primary driving force behind the emergence of new Omicron subvariants [79,80]. Our computational studies of the Omicron variants that exhibit RBD binding with ACE2 showed favorable binding contributions provided by RBD residues K378, R403, K424, K440, K444, K460, N477, and K478, established through the strong electrostatic interactions mediated by lysine residues, which is the result of the Omicron evolution leading to a significant accumulation of positively charged substitutions that interact with the negatively charged ACE2 binding interface [64,81]. By elucidating the roles of convergent evolution, epistatic interactions, electrostatic forces, and allosteric regulation, computational studies have deepened our understanding of how Omicron variants achieve a balance between ACE2 binding and immune evasion.

**Figure 1:**
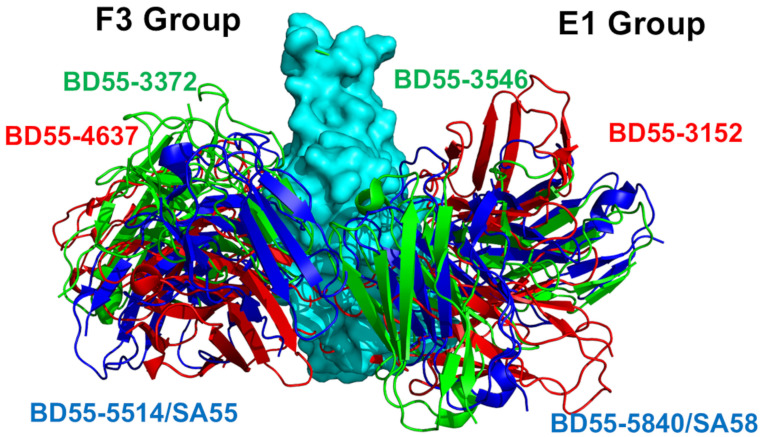
Structural organization of the SARS-CoV-2-RBD complexes with the E1 and F3 groups of Abs. Structural alignment of the E1 and F3 Abs. The S-RBD structure is shown by the cyan surface. The F3 Abs are BD55-3372, pdb id 7WRO (represented by green ribbons), BD55-4637, 7WRJ (represented by red ribbons), and BD55-5514/SA555 pdb id 7Y0W (represented by blue ribbons). The corresponding E1 Abs are BD55-3546, pdb id 7WRY (represented by green ribbons), BD55-3152, pdb id 8WR8 (represented by red ribbons), and BD55-5840/SA58, pdb id 7Y0W (represented by blue ribbons). For clarity of presentation, the heavy and light chains for the corresponding Abs are shown in the same color.

The recently emerged, broadly neutralizing Abs from groups E1 and F3 appeared to target unique RBD epitopes that encompass the RBD stability regions associated with critical spike function and RBM residues that are not essential for ACE2 binding affinity. Combined, these factors may enable the broad immunity escape of these Abs. Experimental and computational studies have suggested that the cross-neutralization Ab activity against Omicron variants may be driven by the balance and tradeoff between multiple energetic factors and these interactions may contribute to the evolution of the escape hotspots involved in antigenic drift and convergent evolution. However, the dynamic and energetic details quantifying the balance and contribution of these factors, particularly the balancing nature of the specific interactions between Abs and the epitope hotspot residues are less well-characterized. In this study, we employ a multifaceted computational and experimental approach to investigate the molecular mechanisms underlying immune evasion by SARS-CoV-2 variants, particularly focusing on the Omicron variant and its interactions with two distinct groups of broadly neutralizing Abs: the E1 group Abs (BD55-3152, BD55-3546, and SA58) and F3 group Abs (BD55-3372, BD55-4637, and SA55) (Figure 1; Supporting Information Table S1).

By constructing dynamic ensembles of the S-Ab complexes, combined with a systematic mutational scanning of the S-RBD residues, we characterize the patterns of mutational sensitivity and generate detailed mutational scanning heatmaps. These heatmaps allow us to identify key escape hotspot centers—regions within the RBD that are prone to mutations, enabling viral escape from antibody neutralization. To rigorously quantify the binding affinities of the S-Ab complexes, we utilized the Molecular Mechanics/Generalized Born Surface Area (MM-GBSA) approach. This method provides precise binding energy calculations and enables residue-based energy decomposition, offering insights into the contributions of individual amino acids to the overall binding stability. Through these computational techniques, we explored the energetic determinants that allow broadly potent antibodies to maintain their efficacy against the RBD sites of Omicron mutations, despite the virus’s evolving immune-evasion strategies. Our findings reveal that two distinct groups of antibodies, E1 and F3 (as depicted in Figure 1), target specific binding epitopes on the RBD. These antibodies primarily rely on strong hydrophobic interactions with critical binding epitope hotspots that are essential for RBD stability and ACE2 receptor binding. In contrast, escape mutations tend to emerge at sites characterized by a combination of strong hydrophobic and electrostatic interactions. This suggests that while hydrophobic interactions are crucial for antibody binding, the interplay between hydrophobic and electrostatic forces may create vulnerabilities that the virus exploits to evade immune detection.

The results of this study provide a framework for rationalizing the roles of hydrophobic and electrostatic interactions in antibody binding and immune evasion. Specifically, we highlight the importance of balancing these interactions to optimize antibody engineering. By exploiting electrostatic complementarity to RBD target sites, it may be possible to design negatively charged neutralizing antibodies with high affinity that target conserved epitopes. Such antibodies could mitigate resistance by avoiding unfavorable interfaces with positively charged RBD residues, thereby enhancing their therapeutic potential. Furthermore, our analysis suggests that RBD mutations and their associated immune escape mechanisms may have reached a level of stability in recently emerged viral lineages. These lineages exhibit greater diversity in the composition of ionizable amino acids, indicating that evolutionary pressures are now leading to epistatic effects and localized electrostatic changes to further enhance immune evasion. This evolutionary trajectory is constrained by the need to maintain RBD stability and ACE2 binding, which limits the scope of possible mutations but also drives the virus to optimize its escape strategies through subtle structural and electrostatic adjustments. By targeting binding hotspots and allosteric communication centers within the RBD, it may be possible to design interventions that not only neutralize the virus but also reduce the likelihood of drug resistance. These findings underscore the importance of understanding the intricate balance between hydrophobic and electrostatic interactions in antibody design and highlight potential avenues for the development of next-generation therapeutics aimed at combating emerging SARS-CoV-2 variants.

## 2. Materials and Methods

### 2.1. Molecular Dynamics Simulations

The crystal and cryo-EM structures of the Omicron RBD-Ab complexes were obtained from the Protein Data Bank [82]. All-atom MD simulations were performed for the E1 group Abs (BD55-3546 Fab bound to SARS-COV2 Delta RBD complex, pdb id 7WRY; BD55-3152 Fab bound to Omicron BA.1, pdb id 7WR8, and SA58 bound to Omicron BA.1, pdb id 7Y0W) and F3 Abs (BD55-3372 bound to SARS-CoV-2 delta RBD, pdb id 7WRO, BD55-4637 Fab bound to Omicron BA.1, pdb id 7WRJ, and SA55 bound to Omicron BA.1, pdb id 7Y0W). For simulated structures, hydrogen atoms and missing residues were initially added and assigned according to the WHATIF program web interface [83]. The missing regions were reconstructed and optimized using a template-based loop prediction approach ArchPRED [84]. The side chain rotamers were refined and optimized using the SCWRL4, the version number is SCWRL4.0.2 tool [85]. The protonation states for all the titratable residues of the Ab and RBD proteins were predicted at pH 7.0 using Propka 3.1 software and web server [86,87]. The protein structures were then optimized using atomic-level energy minimization with composite physics and knowledge-based force fields implemented in the 3Drefine method [88,89]. We considered glycans that were resolved in the structures. The NAMD 2.13-multicore-CUDA package [90] with CHARMM36 force field [91] was employed to perform 1µs all-atom MD simulations for the RBD-Ab complexes. The structures of the complexes were prepared using Visual Molecular Dynamics (VMD 1.9.3) [92] and the CHARMM-GUI web server [93,94] using the Solutions Builder tool, accessed through the CHARMM-GUI interface, is version 1.9. Hydrogen atoms were modeled onto the structures prior to solvation with TIP3P water molecules [95] in a periodic box that extended 10 Å beyond any protein atom in the system. To neutralize the biological system before the simulation, Na+ and Cl− ions were added in physiological concentrations to achieve charge neutrality, and a salt concentration of 150 mM of NaCl was used to mimic the physiological concentration. All Na+ and Cl− ions were placed at least 8 Å away from any protein atoms and from each other. MD simulations are typically performed in an aqueous environment in which the number of ions remains fixed for the duration of the simulation, with a minimally neutralizing ion environment or salt pairs to match the macroscopic salt concentration [96].

All protein systems were subjected to a minimization protocol consisting of two stages. First, minimization was performed for 100,000 steps with all the hydrogen-containing bonds constrained and the protein atoms fixed. In the second stage, minimization was performed for 50,000 steps with all the protein backbone atoms fixed and for an additional 10,000 steps with no fixed atoms. After minimization, the protein systems were equilibrated in steps by gradually increasing the system temperature in steps of 20 K, from 10 K to 310 K, and at each step, a 1 ns equilibration was performed, maintaining a restraint of 10 kcal mol^−1^ Å^−2^ on the protein Cα atoms. After the restraints on the protein atoms were removed, the system was equilibrated for an additional 10 ns. Long-range, non-bonded van der Waals interactions were computed using an atom-based cutoff of 12 Å, with the switching function beginning at 10 Å and reaching zero at 14 Å. The SHAKE method was used to constrain all the bonds associated with hydrogen atoms.

The simulations were run using a leap-frog integrator with a 2 fs integration time step. The ShakeH algorithm in NAMD was applied for the water molecule constraints. The long-range electrostatic interactions were calculated using the particle mesh Ewald method [97] with a cut-off of 1.0 nm and a fourth-order (cubic) interpolation. The simulations were performed under an NPT ensemble with a Langevin thermostat and a Nosé–Hoover Langevin piston at 310 K and 1 atm. The damping coefficient (gamma) of the Langevin thermostat was 1/ps. In NAMD, the Nosé–Hoover Langevin piston method is a combination of the Nosé–Hoover constant pressure method [98] and piston fluctuation control implemented using Langevin dynamics [99,100]. An NPT production simulation was run on equilibrated structures for 1 µs, keeping the temperature at 310 K and using constant pressure (1 atm).

### 2.2. Binding Free Energy Computations: Mutational Scanning and Sensitivity Analysis

To understand the molecular mechanisms underlying the interactions between the SARS-CoV-2 S-RBD and neutralizing Abs, we conducted a comprehensive mutational scanning analysis of the binding epitope residues. This approach systematically evaluated the effects of mutations on protein stability and binding free energy, providing insights into the structural and energetic determinants of the RBD-Ab interactions. Each binding epitope residue in the RBD-Ab complexes was systematically mutated using all possible amino acid substitutions. The corresponding changes in protein stability and binding free energy were computed using the BeAtMuSiC approach [101,102,103]. This method relies on statistical potential that describe pairwise inter-residue distances, backbone torsion angles, and solvent accessibility. The BeAtMuSiC approach evaluates the impact of mutations on both the strength of interactions at the protein–protein interface and the overall stability of the complex.

The binding free energy of a protein–protein complex is expressed as the difference between the folding free energy of the complex and the folding free energies of the individual binding partners:

(1) $$\Delta G_{bind}=G^{com}-G^{A}-G^{B}$$

The change in the binding energy due to a mutation was calculated then as follows:

(2) $$\Delta \Delta G_{bind}=\Delta G_{bind}^{mut}-\Delta G_{bind}^{wt}$$

To ensure the robustness of our results, we leveraged rapid calculations based on statistical potentials, computing ensemble-averaged binding free energy changes using equilibrium samples from molecular dynamics (MD) simulation trajectories. The binding free energy changes were averaged over 10,000 equilibrium samples for each system studied. We used 1000 ns of equilibrated trajectory data for each system, with snapshots collected at 100 ps intervals. This approach provided a total of 10,000 frames per system for binding free energy calculations.

### 2.3. Binding Free Energy Computations

For the binding free energy calculations, we employed the Molecular Mechanics/Generalized Born Surface Area (MM/GBSA) method [104,105]. To ensure robust sampling, equilibrium trajectories were extracted from the production phase of the MD simulations. Specifically, we used 1000 ns of equilibrated trajectory data for each system, with snapshots collected at 100 ps intervals. This approach provided a total of 10,000 frames per system for the binding free energy calculations. Additionally, we conducted an energy decomposition analysis to evaluate the contribution of each amino acid during the binding of RBD to Abs [106,107].

The binding free energy for the RBD-Ab complex was obtained using the following equations:

(3) $${\Delta G}_{\mathrm{bind}}=G_{\mathrm{RBD}-\mathrm{AB}}-G_{\mathrm{RBD}}-G_{\mathrm{AB}}$$

(4) $$\Delta G_{bind,MMGBSA}=\Delta E_{MM}+\Delta G_{sol}-T\Delta S$$

where ΔE_MM_ is the total gas phase energy (sum of ΔEinternal, ΔEelectrostatic, and ΔEvdw); ΔGsol is the sum of polar (ΔGGB) and non-polar (ΔGSA) contributions to solvation. Here, GRBD–AB represent the average, calculated using the snapshots of a single trajectory of the complex; GRBD and GAB correspond to the free energy of RBD and Ab proteins, respectively.

The polar and non-polar contributions to the solvation free energy were calculated using a Generalized Born solvent model, considering the solvent-accessible surface area [108]. MM-GBSA was employed to predict the binding free energy and determine the free energy contributions to the binding free energy of a protein–protein complex on a per-residue basis. The binding free energy with MM-GBSA was computed by averaging the results of computations over 10,000 samples from the equilibrium ensembles. We used 1000 ns of equilibrated trajectory data for each system, with snapshots collected at 100 ps intervals. This approach provided a total of 10,000 frames per system for binding free energy calculations.

Two computational protocols were considered. The single-trajectory protocol uses one trajectory of the RBD-Ab complex, reducing noise by canceling out the contributions of intermolecular energy. This protocol is suitable when significant structural changes upon binding are not expected. The separate trajectory protocol can utilize three separate trajectories for the complex, RBD, and Ab, which is more appropriate for systems with large conformational changes between unbound and bound proteins. In this study, we employed a single-trajectory protocol due to its lower noise and applicability to systems with minimal structural reorganization upon binding. The contributions of entropy are typically excluded from the calculations because the entropic differences in relative binding affinities are expected to be small, with only minor mutational changes [109,110]. However, for the absolute affinities, the entropy term is needed, owing to the loss of translational and rotational freedom when the ligand binds. To account for the dynamic nature of the RBD and its potential entropic contributions, we performed a normal mode analysis (NMA) to estimate the entropy changes. Entropy calculations were conducted on a subset of 500 evenly spaced snapshots extracted from the equilibrated trajectories. The MM/GBSA calculations were performed using the MMPBSA.py module in AMBER, with the dielectric constant set to 1 for the solute and 80 for the solvent in the AmberTools21 package [111] and gmx_MMPBSA, the version number of AmberTools is 21. gmx_MMPBSA version number is 5.5, a new tool to perform end-state free energy calculations from the CHARMM and GROMACS trajectories [112].

To further dissect the binding interactions, we conducted an energy decomposition analysis, evaluating the contribution of each amino acid residue to the binding free energy. This analysis revealed key residues that stabilize or destabilize the RBD-Ab complex, highlighting potential targets for therapeutic intervention. In summary, mutational scanning and binding free energy analysis enable detailed quantitative characterization of the molecular interactions between the SARS-CoV-2 RBD and neutralizing Abs. By combining the BeAtMuSiC approach with MM-GBSA calculations, we identified critical residues and energetic contributions that govern binding affinity and stability. The computational protocols and tools employed in this study offer a robust framework for analyzing protein–protein interactions.

## 3. Results

### 3.1. Evolutionary Survey and Analysis of Omicron JN.1 Lineages

**Figure 2:**
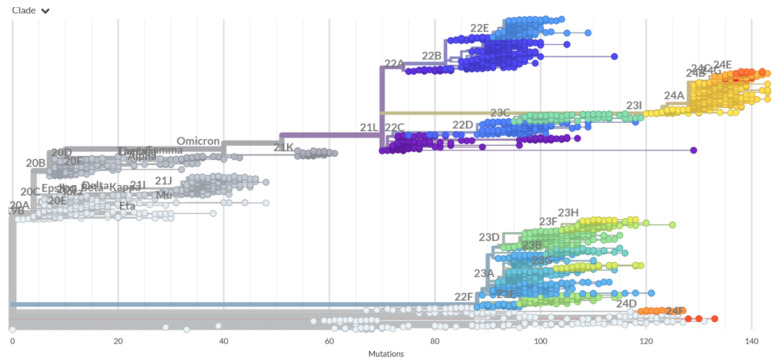
Phylogenetic relationships of existing SARS-CoV-2 clades. An overview of the phylogenetic analysis and SARS-CoV-2 clade classification highlights the evolution of the SARSB-CoV-2 lineages using a rectangular phylogenetic tree representation. The graph was generated using Nextstrain, an open-source project for the real-time tracking of evolving pathogen populations (https://nextstrain.org/, (accessed on 19 December 2024)).

We first examined the evolutionary traits of SARS-CoV-2 lineages, particularly among the Omicron variants that are illustrated by the phylogenetic analysis, using their corresponding clades’ nomenclature from Nextstrain, an open-source project for the real-time tracking of evolving pathogen populations (https://nextstrain.org/, (accessed on 19 December 2024)) [113]. Nextstrain provides dynamic and interactive visualizations of the phylogenetic tree of SARS-CoV-2, enabling exploration and illustration of the evolutionary relationships between different lineages and variants. An overview of the phylogenetic analysis and SARS-CoV-2 clade classification (Figure 2, Supporting Information, Figure S1) highlights the evolution of SARSB-CoV-2 lineages. Clades 19A and 19B are ancestor lineages that emerged in Wuhan. Clades 20D to 20J include Alpha (lineage B.1.1.7), Beta (lineage B.1.351), and Gamma (lineage P.1). Clades 21A to 21J include Delta, Lambda (lineage C.37), Mu (lineage B.1.621), and Epsilon (lineages B.1.429). Clades 21K (BA.1) and 21L (BA.2) are the Omicron sublineages that emerged from strain 21M (lineage B.1.1.529).

JN.1 is a variant of BA.2.86 that emerged independently of Omicron BA.2 and received Nextstrain clade classification 24A (Figure 2). JN.1 diversified into a number of sublineages that share recurrent mutations R346T (JN.1.18), F456L (JN.1.16), T572I (JN.1.7), or combinations of these mutations (KP.2: JN.1 + S:R346T, S:F456L, S:V1104L), and was designated Nextstrain clade 24B. KP.3 (JN.1.11.1.3), featured mutations R346T, L455S, F456L, Q493E, and V1104L. KP.3 (JN.1 + S:F456L, S:Q493E, S:V1104L), and received the classification of Nextstrain clade 24C. Clade 24G, lineage KP.2.3, is a descendant of clade 24B with extra spike substitutions, S:R346T and S:H146Q, and the deletion of S:S31- and ORF3a:K67N (https://github.com/nextstrain/ncov/pull/1152, (accessed on 19 December 2024)). Clade 24D is an XDV.1 variant and clade 24E is a KP.3.1.1 variant (Supporting Information, Figure S1A–C).

The most recently designated clade 24F of lineage XEC resulted from a recombination event of KS.1.1 and KP.3.3. KS.1.1 belongs to clade 24A (lineage JN.1) and KP.3.3 belongs to clade 24C (lineage KP.3), which itself descends from JN.1. Hence, XEC is a recombinant of JN.1-derived diversity. According to the Coronavirus Network (CoViNet) (https://data.who.int/dashboards/covid19/variants, (accessed on 19 December 2024), the currently circulating VOIs are JN.1.7 (JN.1 + S:T572I, S:E1150D clade 24A), KP.2 (JN.1 + S:R346T, S:F456L, S:V1104L, clade 24B). KP.3 (JN.1 + S:F456L, S:Q493E, S:V1104L, clade 24C), KP.3.1.1 (KP.3 + S:S31-, clade 24C), JN.1.18 (JN.1 + S:R346T, clade 24A), LB.1 (JN.1 + S:S31-, S:Q183H, S:R346T, S:F456L, clade 24A), and XEC (JN.1 + S:T22N, S:F59S, S:F456L, S:Q493E, S:V1104L, clade 24F) (Figure 1). The current evolutionary divergences between the XBB and JN.1 lineages illustrated by the Nextstrain diagrams (Supporting Information, Figure S1C,D) indicate that the evolutionary trajectories of the Omicron lineages can proceed through complex recombination, antigenic drift and convergent evolution. The recent data on growth advantage relative to population average in the US showed that XEC and KP.3.1.1 are the most common Pango lineage and have the highest fitness relative to the population average (https://github.com/nextstrain/ncov/pull/1152, (accessed on 19 December 2024)) (Supporting Information, Figure S1D).

According to the WHO Coronavirus Network (CoViNet) (https://data.who.int/dashboards/covid19/variants and https://data.who.int/dashboards/covid19/circulation?n=o), (accessed on 19 December 2024) who, in recent months, conducted an analysis of the prevalence of SARS-CoV-2 variants of interest (VOI) and variants under monitoring (VUM), the dominant variants are KP.3.1.1 (prevalence: 53.59%, VUM), XEC (prevalence: 26.14%, VUM) JN.1 (prevalence: 9.8%, VOI), KP.3 ((JN.1 + S:F456L, S:Q493E, S:V1104L; prevalence: 4.14%, VUM), KP.2 (prevalence: 1.53%, VUM), JN.1.18 ((JN.1 + S:R346T; prevalence: 0.87%, VUM), and BA.2.86 (prevalence: 0.22%, VOI). As of December 2024, the currently circulating variants in the US are predominantly KP.3.1.1 (32.6%), XEC (19.5%), MC.16 (3.4%), MC.13 (2.7%), NL.2 (2.6%), MC.1 (2.2%), MC.10.1 (2%), XEC.2 (1.6%), MC.11 (1.5%), KP.2.3 (1.4%), LB.1.3.1 (1.4%), and XEK (1.3%) (https://public.tableau.com/app/profile/raj.rajnarayanan/viz/USAVariantDB/VariantDashboard, (accessed on 19 December 2024)). According to Cov-Spectrum data (https://cov-spectrum.org/explore/World/AllSamples/, (accessed on 19 December 2024)), the JN.1 variant accounted for 65.4% of the samples collected and analyzed within the specified date range (3 June 2024 to 26 November 2024). This percentage represents the high prevalence of the JN.1 variant in the global samples tested during that period (Supporting Information, Figure S2). It is instructive to notice that a high proportion of the global samples collected and analyzed during this latest period are dominated by other JN.1 descendants; specifically 54.8% for KP.3, 49.2% for KP.3.1.1, and 31.5% for the XEC variant (Supporting Information, Figure S2). Notice that the sum of percentages for the variants, presented on Cov-Spectrum, can exceed 100% because some samples may contain multiple variants. The Cov-Spectrum comparison showed the prevalence of different variants across various regions and helps to visualize how specific variants are spreading globally and to identify regional differences in variant distribution (Supporting Information, Figure S2). The proportion of sequences over the last six months showed a general decline for the JN.1, FLiRT, and KP.3 variants (Supporting Information, Figure S2). The current lineage frequency is considerably increased for the presently dominant XEC variant (Supporting Information, Figure S2). KP.3.1.1 is KP.3; the additionally convergently acquired S31 deletion have emerged as an advantageous mutation and currently demonstrates high lineage frequency (Supporting Information, Figures S1D and S2).

A phylogenetic analysis of the latest SARS-CoV-2 clades (JN.1, KP.2, KP.3) also revealed the emergence of recurrent mutations that appeared to play a critical role in modulating binding with ACE2 and Abs. Notably, convergent evolution mutations such as F456L, F486P, and Q493E were observed across multiple clades and have been shown to play a critical role in antibody binding and ACE2 affinity across various functional studies [40,41,42,43,44,45,46,47,48]. These mutations represent key evolutionary adaptations that align with the functional hotspots identified in the binding studies, revealing the epistatic effects of mutations Q493E and F456L on ACE2 binding [47,48]. Structural studies also showed that the Omicron subvariants sharing an F456L mutation typically show a greatly increased Ab evasion ability, with the most recent SA55 Ab displaying potency against these convergent mutans [57]. These findings highlighted the significance of convergent evolution in shaping the binding properties of the S protein. In the following sections, we present a computational mutational profiling of the S-RBD binding with a panel of neutralizing Abs. Through integrating phylogenetic trends with biophysical insights, we will examine the mechanisms of how evolutionary pressures drive the emergence of functionally optimized variants that can effectively evade the binding to Abs.

### 3.2. Structural Analysis of the RBD Complexes with E1 and F3 Group Abs

We began with a structural analysis of the S-RBD binding with the two classes of Abs E and F [54,55,56,57,58]. Groups E and F are categorized into E1–E3 and F1–F3, covering the front and back of the RBD. We specifically focused on two groups: E1 group Abs (BD55-3546 Fab, bound to the SARS-COV2 Delta RBD complex, pdb id 7WRY; BD55-3152 Fab, bound to Omicron BA.1, pdb id 7WR8; and SA58, bound to Omicron BA.1, pdb id 7Y0W) and F3 Abs (BD55-3372, bound to SARS-CoV-2 delta RBD, pdb id 7WRO; BD55-4637 Fab, bound to Omicron BA.1, pdb id 7WRJ; and SA55, bound to Omicron BA.1, pdb id 7Y0W) (Figure 1). These two groups of Abs emerged as a result of the high-throughput epitope determination and selection of broadly neutralizing Abs that target rare RBD epitopes that are associated with critical functions, such as ACE2 binding and RBD stability, to avoid immunity-directed escape mutations [54,57].

**Figure 3:**
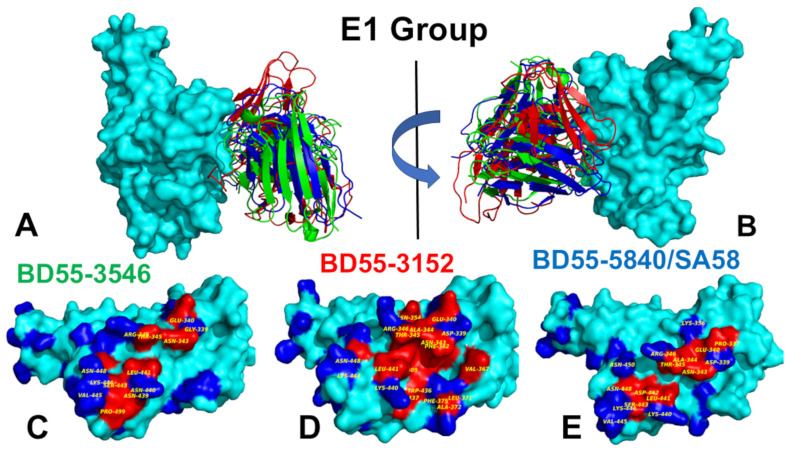
Structural organization of the RBD complexes and binding epitopes of the E1 group of Abs. (**A**,**B**) The two views of the RBD-Ab complexes. The S-RBD structure is shown as the cyan surface. The E1 Abs are BD55-3546, pdb id 7WRY (in green ribbons), BD55-3152, pdb id 8WR8 (in red ribbons), and BD55-5840/SA58, pdb id 7Y0W (in blue ribbons). For clarity of presentation, the heavy and light chains for the corresponding Abs are shown in the same color. (**C**) The RBD surface and binding epitope for BD55-3546. RBD is shown as the cyan surface. The binding epitope residues are represented by the red surface (G339, E340, V341, N343, A344, T345, R346, F347, K356, N439, N440, L441, D442, S443, K444, V445, N448, N450, Y41, P499, R509). (**D**) The RBD surface and binding epitope for BD55-3152. RBD is shown as a cyan surface. The binding epitope residues are represented by the red surface (D339, E340, V341, F342, N343, A344, T345, R346, F347, N354, V367, L368, L371, F375, W436, N437, S438, K440, L441, D442, K444, N448, Y451, R509). (**E**) The RBD surface and binding epitope for SA58. RBD is shown as a cyan surface. The binding epitope residues are represented by the red surface (P337 D339, E340, V341, N343, A344, T345, R346, K356, R357, I358, K440, L441, D442, S443, K444, V445, N448, N450, Y451, R509). The sites of Omicron XBB, BA.2.86, and JN.1 lineages are shown on panels (**C**–**E**) as the blue surface (i.e., residues 339, 346, 356, 371, 373, 375 376, 403, 405, 408, 417, 440, 444, 445, 446, 450, 452, 455, 456, 460, 475, 477, 478, 481, 484, 486, 493, 498, 501, 505). Binding epitope residues are defined as the RBD binding interface residues that directly interact with antibodies. We used the BeAtMuSiC approach [101,102,103] for the contact predictor to identify binding interface residues. Residues are considered part of the interface if they are within a defined cutoff distance (typically 5 Å) from atoms in the binding partner. Our definition of binding interface residues corresponds to the definition of the contact residues in the experimental studies.

The E1 group of Abs binds to the RBD (Figure 3) and is known to be susceptible to mutations of T345 and R346 [54,57]. BD55-3546 binds to the binding epitope consisting of residues 340–347, 440–451 and P499 (Figure 3, Table S2). The topology of the binding epitope reveals two major patches: one corresponds to residues 340–347 and 356, while the second separate patch is formed of residues 440–451 and 499 (Figure 3). This Ab makes extensive contact with the large number of the RBD residues, where continuous stretches of the binding epitope are formed by residues 340–347 and especially 440–451 (Figure 3). Importantly, we found a large number of contacts between T345 and the R105, W94, Y103, L96, Y91, and N92 Ab residues. Fewer contacts were made with the R346 position. R346 is particularly interesting as the FLiRT variant has an additional R346T mutation in the SLip backbone. The R346T mutation is featured in KP.2 (JN.1 + S:R346T, S:F456L, S:V1104L), LB.1 (JN.1 + S:S31-, S:Q183H, S:R346T, S:F456L), and KP.2.3 variants but conspicuously absent in the KP.3 (JN.1 + S:F456L, S:Q493E, S:V1104L) and XEC variants (JN.1 + S:T22N, S:F59S, S:F456L, S:Q493E, S:V1104L). Another significant binding residue, N440, is located in a pocket formed by the heavy chain residues W50, N52, T55, I57, P58, and T59, leading to extensive van der Waals and hydrogen-bond interactions with the Ab (Figure 3, Table S2). Another important hotspot, K444, is involved in contacts with the heavy chain’s N31, N54, and L102 positions (Figure 3, Table S2). Interestingly, while most E1 Abs are sensitive to mutations of T345 and R346, BD55-3546 mainly escaped through the T345 and N440 mutations [57]. It appears that these RBD positions formed the most contacts with BD55-3546 and are engaged in the interaction networks.

The BD55-3152 targets a similar epitope that is more contiguous and even slightly larger than the one for BD55-3546 (Figure 3, Table S3). The epitope includes RBD residue regions 339–347, 354; 367–375; 436–448; and P509 (Figure 3). Hence, the BD55-3152 epitope includes additional residues 367–375, which overlap with the positions of Omicron mutations S371P and S373F. A structural analysis indicated that the densest contact networks for BD5-3152 with the RBD are formed by N343, A344, T345, and R346 (Figure 3, Table S3). T345 makes 10 particularly strong contacts with Y33, Y31, G110, S111, P112, and L113 of the Ab (Table S2). Similarly, R346 is involved in multiple contacts with the Ab, particularly Q30 and D50 of the light chain (Table S3).

For SA58, the binding epitope is generally very similar, and includes residues 337-346, 356, and residues 440–450 (Figure 3, Table S4). A structural analysis showed that most contacts are made by the E340, T345, and R346 RBD sites (Table S4). T345 is enclosed by Y93, L98, S94, N95, Y105, L94, and W96, and mutations in this position result in immune escape [57]. Another sensitive site is K444, which interacts with T30, N32, S31, D54, and Y102 of the SA58 Ab (Table S4). According to the experimental data, neutralization via SA58 is affected by T345N, R346Q, R346T, E340D, and the E340K and K444E mutations escape neutralization [57]. Group E1 Abs are generally sensitive to the changes in G339, E340, T345, and especially R346, as revealed by their escaping mutation profiles [54,57]. According to the escape calculations made using the updated data, the escape positions are indeed T345 and R346 [46]. In contrast to R346K, R346S/T could greatly compromise the binding activities of E1 Abs [57]. We also highlighted the sites of XBB, BA.2.86, and JN.1 lineages; these are displayed as a blue surface (i.e., residues 339, 346, 356, 371, 373, 375 376, 403, 405, 408, 417, 440, 444, 445, 446, 450, 452, 455, 456, 460, 475, 477, 478, 481, 484, 486, 493, 498, 501, and 505) (Figure 3C,E). This structural mapping clearly shows the overlap between the binding epitope and the sites of Omicron mutations.

**Figure 4:**
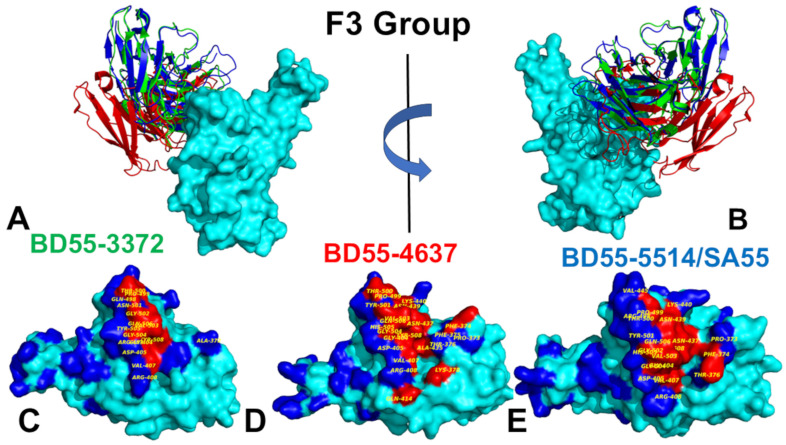
Structural organization of the RBD complexes and binding epitopes of the F3 group of Abs. (**A**,**B**) The two views of the RBD-Ab complexes. The S-RBD structure is shown as the cyan surface. The E1 Abs are BD55-3372, pdb id 7WRO (in green ribbons), BD55-4637, pdb id 7WRJ (in red ribbons), and BD55-5514/SA55, pdb id 7Y0W (in blue ribbons). For clarity of presentation, the heavy and light chains for the corresponding Abs are shown in the same color. (**C**) The RBD surface and binding epitope for BD55-3372. RBD is shown as a cyan surface. The binding epitope residues are represented by the red surface (A372, T403, G404, D405, E406, V407, R408, Q409, N437, N439, Q498, P499, T500, N501, G50, V503, G504, Y505, Q506, Y508). (**D**) The RBD surface and binding epitope for BD55-4637. RBD is shown as a cyan surface. The binding epitope residues are represented by the red surface (A37, P373, F374, F375, T376, F377, K378, R403, G404, D405, V407, R408, Q414, V433, A435, 436, N437, N439, K440, S496, R498, T500, Y501, G502, V503, G504, H505, Q506, Y508). (**E**) The RBD surface and binding epitope for SA55. RBD is shown as a cyan surface. The binding epitope residues are represented by the red surface (P373, F374, T376, R403, G404, D405, E406, V407, R408, N437, N439, K440, V445, Y495, 496, R498, P499, T500, Y501, G502, V503, G504, H505, Q506, Y508). The sites of Omicron XBB, BA.2.86, and JN.1 lineages are shown as the blue surface (residues 339, 346, 356, 371, 373, 375 376, 403, 405, 408, 417, 440, 444, 445, 446, 450, 452, 455, 456, 460, 475, 477, 478, 481, 484, 486, 493, 498, 501, 505). Binding epitope residues are defined as the RBD binding interface residues that directly interact with antibodies. We used the BeAtMuSiC approach [101,102,103] contact predictor to identify binding interface residues. Residues are considered part of the interface if they are within a defined cutoff distance of 5 Å from atoms in the binding partner. Our definition of binding interface residues corresponds to the definition of the contact residues in the experimental studies.

The F3 group BD55-3372 Ab binds to the conserved epitope formed by residues 372–375 and 404–408, as well as with RBD segments 498–509, which are critical for ACE2 binding (Figure 4, Table S5). BD55-3372 exhibited high neutralization potency compared to other Abs in Group F3 [54]. BD55-4637 has a much larger binding epitope and forms stable contacts with residues 374–378, 403–408, conserved stable residues 436–440, and ACE2 binding sites 500–508 (Figure 4, Table S6). BD55-4637 forms most of its contacts with the RBD residues that are critical for RBD stability and ACE2 binding, while it has fewer interactions with R403, D405, and R408 (Figure 4, Table S6). We also found that BD55-4637 makes multiple contacts with N439.

The structural analysis of the binding epitope for SA55 Ab showed RBD positions 373–376, 404–408, 436–440, 445, 446, and an extended RBD segment of residues 498–508 (Figure 4, Table S7). SA55 targets segments 498–508, which are critical for RBD functions, making multiple contacts with Y501, G502, V503, G504, H505, and T508 (Figure 4, Table S7). The core of the binding epitope corresponds to the conserved RBD region (residues 436–440), which is critical for RBD stability, and ACE2-binding segments 498–508, where residues are unlikely to mutate to escape Abs as they are critical to the ACE2 affinity. This segment includes Y501 and H505, which are energetic hotspots for ACE2 binding where the Ab competes with the host receptor to bind with the S-RBD, and as a result, these positions cannot be exploited by the virus to evolve Ab-resistant mutations. However, there are a number of Omicron mutational sites, such as S373P and S375F, that interact with L94 and F55 of the heavy chain, where Omicron mutations could potentially alter the binding with the Ab (Figure 4, Table S7). The structural analysis also revealed multiple favorable contacts between SA55 and T376 using F55 of the heavy chain and interactions with D405 using L54, S31, T28, and R30 of the heavy chain. Although T376, D405, and R408 are involved in the interaction with SA55, these residues are all located closer to the periphery of the binding epitope and some of the mutations in these positions may be tolerated. Indeed, according to the illuminating study by Cao and colleagues, SA55 displayed high potency against the Omicron subvariants, sharing F456L mutations including HV.1 (L452R + F456L) and JD.1.1(L455F/F456L + A475V), which typically greatly increase the Ab evasion ability in other Abs [57]. Nonetheless, the binding and neutralization activity of SA55 may be compromised by Y508H, G504S, K440E, and V503E and G504D may escape the effects [57].

Although a structural analysis of the binding epitope and contacts alone is obviously not sufficient for the prediction of escape hotspots and an in-depth energetic assessment is required, the topological composition of the binding epitope revealed that positions V503 and G504, which are involved in many contacts with the Ab (Figure 4, Table S7), could be vulnerable to escaping mutations. Indeed, we found that V503 makes multiple hydrophobic contacts with L54, P101, I52, and S31 while G504 is involved in interactions with L54, H32, S31, and R30 of the heavy chain (Figure 4, Table S7). In this context, it is not unexpected that changing the chemical identity of V503 to V503E and G504 to G504D could disrupt the interaction network and cause immune escape. In the next sections, we perform a detailed energetic analysis using full mutational scanning and MM-GBSA binding free energy calculations to dissect the effect of specific changes and the contributions of the RBD residues to the binding affinity with E1 and F3 Abs.

### 3.3. MD Simulations of the S-RBD Complexes with Abs Reveal Specific Patterns of Moderate RBD Mobility

All atom MD simulations were performed for the S-RBD complexes using a panel of studied Abs to examine the conformational landscapes and determine the specific dynamic signatures induced by Abs. The primary focus of this study was on the dynamics and energetic contributions of RBD residues, but the flexibility of the Ab residues, particularly in the complementarity-determining regions (CDRs), could also play a role in shaping antibody–RBD interactions. However, the Ab proteins are generally more rigid compared to the RBD, and their dynamics were not the central focus of this work. To streamline the presentation and analysis of our results and the major findings, we provide a detailed analysis of the conformational ensembles of the RBD protein and elucidate the effects of RBD mutations on the dynamics and molecular determinants of RBD-Ab binding, which is the key driver of viral evolution

**Figure 5:**
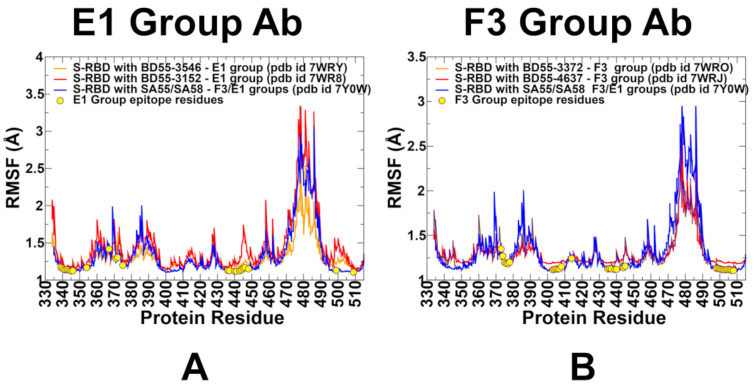
Conformational dynamics profiles obtained from simulations of the RBD-Ab complexes. (**A**) The RMSF profiles for the RBD residues obtained from MD simulations of the S-RBD complexes with E1 group Abs: BD55-3546 (orange lines), BD55-3152 (red lines), and BD55-5840/SA58, pdb id 7Y0W (blue lines). (**B**) The RMSF profiles for the RBD residues obtained from MD simulations of the S-RBD complexes with the F3 group Abs: BD55-3372 (green lines), BD55-4637, 7WRJ (red lines), and BD55-5514/SA555, pdb id 7Y0W (blue ribbons). The positions of the binding epitope residues for E1 and F3 group of Abs are highlighted by the yellow filled circles in the panels (**A**,**B**).

To characterize the dynamic flexibility of the RBD in a complex with different antibody groups, we performed a root-mean-square fluctuation (RMSF) analysis of the equilibrated MD trajectories (Figure 5). Overall, the RMSF profiles of the RBD were fairly similar across the antibody complexes, reflecting the conserved structural stability of the RBD core. However, notable differences were observed in specific regions, particularly in the 470–490 loop, which exhibited higher flexibility in certain complexes (Figure 5). This region is known to be a conformationally dynamic loop, and its flexibility likely reflects the conformational adjustments induced by different antibody groups. These localized differences in dynamics provide critical insights into how specific antibodies engage with the RBD and influence their conformational landscape, which may have implications for binding affinity and immune escape mechanisms.

**Figure 6:**
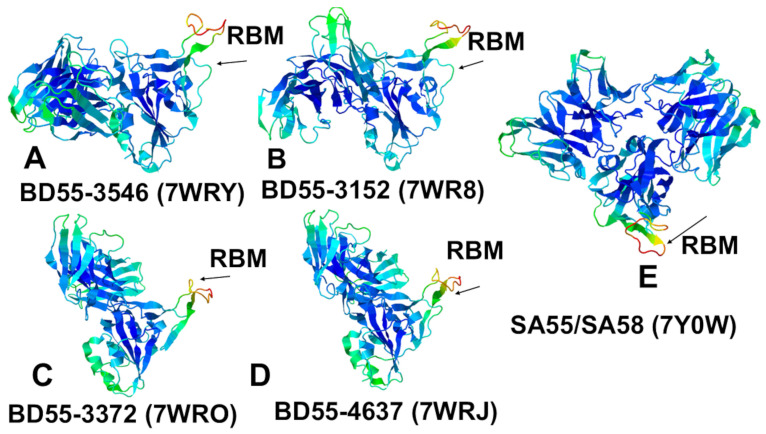
Conformational dynamics profiles projected onto structures of the RBD complexes with E1 group Abs (BD55-3546 (**A**) and BD55-3152 (**B**)) and RBD complexes with F3 group Abs (BD55-3372 (**C**) and BD55-4637 (**D**)). The conformational dynamics profile is projected onto the RBD structure using a combination of SA55 and SA58 Abs (**E**). The structures are colored in a sliding color scale ranging from blue (rigid regions) to red (flexible regions).

Despite notable differences in the binding epitopes for F3 and E1 Abs, the conformational dynamics profiles for these Abs are fairly similar (Figure 5). The conformational mobility distributions for the S-RBD complex with E1 and F3 Abs were characteristic of the RBD minima corresponding to residues 374–377, the RBD core residue cluster (residues 396–403), and residues 445–456 (Figure 5). The RBD core consisted of antiparallel β strands (β1 to β4 and β7) (residues 354–358, 376–380, 394–403, 431–438, 507–516). The β-sheets β5 and β6 (residues 451–454 and 492–495) corresponded to highly stable positions that anchor the RBM region to the central core. For the E1 group, the BD55-3152 targets large epitope formed by RBD regions 339–347, 367–375, and 436–448. The results of the structural analysis showed significant contact with the T345 and R346 positions (Figure 3, Table S3). However, we observed appreciable fluctuations in residues 367–375 and 440–448 in this complex, as well as in positions 458–460 (Figure 5A). In addition, the dynamics profile for this complex exhibited markedly increased RBM mobility (residues 470–490) compared to another Ab BD55-3546 from this group (Figure 5A). Group E1 Abs are also generally sensitive to mutations in G339, E340, T345, and especially R346 [54,57], and according to our dynamics analysis, these RBD positions became largely immobilized for all E1 Abs, displaying only small thermal fluctuations (Figure 5A). Hence, the dynamics profiles indicated that the RBD positions that are vulnerable to escape in this group are associated with the epitope residues that become highly stable due to their multiple points of contact with the Abs, suggesting that mutations in these sites are likely to induce a significant destabilizing effect. By projecting conformational fluctuation profiles onto the structures of the E1 Ab-RBD complexes (Figure 6), we noticed a considerable rigidification of the RBD, with only the RBM region displaying high mobility.

For the F3 Ab complexes, we also found that the binding epitope positions that make contact with the Abs are associated with stabilization of the corresponding regions (Figure 5B). Among the F3 Abs, BD55-4637 exhibited an expanded binding epitope formed by residues 374–378, 403–408, 414, 436–445, and 500–508. Interestingly, residues 439–446 in the complex with BD55-4637 showed some degree of plasticity despite their interactions with the Ab. We also observed that the binding of F3 Abs still leaves some regions of the RBD moderately mobile, including residues 365–385 and the RBM region (Figure 6C,D). At the same time, the RBD residues 400–456 are effectively immobilized in the BD55-4637 complex with the RBD (Figure 5B and Figure 6C,D). Of special importance is the analysis of conformational dynamics profile of the RBD complex with an Ab cocktail of SA55/SA58, in which SA55 belongs to the F3 group and SA58 is in the E1 group (Figure 5 and Figure 6E). We found that binding these two Abs to different sides of the RBD can result in prominent stabilization of the RBD and changes in the RBD mobility, showing moderate fluctuations in regions 380–395 and 455–465 and increasing mobility in the RBM loops (residues 473–487) (Figure 5 and Figure 6E). Notably, the SA55 neutralizing activity remains largely unimpaired upon changes in S375F, R403K, D405N, R408S, N440K, V445H, Q498R, N501Y, and Y505H [57]. The potential positions of immune escape for SA55 are T376, K378, D405, R408, and G504, while the accumulation of mutations on E340D/K, T345P, R346Q, and K444N may affect the neutralization of SA58 [57]. According to the dynamics analysis, these positions belong to the RBD regions that are stabilized by the SA55/SA58 binding. Interestingly, R403, D405, R408, K440, and V445, whose mutations are tolerated by SA55 binding, exhibited small thermal fluctuations (Figure 5). It is possible that for the T376, D405, and R408 sites that are stable and involved in the interactions with SA55, their structural location at the periphery of the SA55 binding epitope may provide them with a certain degree of tolerance to substitutions using conformational plasticity in these positions.

Another important observation of dynamic analysis for SA55/SA58 complex with the RBD is that stable positions implicated in immune escape are immediately adjacent to the RBD with the increased mobility, i.e., potentially susceptible sites of escape may correspond to local hinge sites that modulate movements of the RBD (Figure 5). Of particular notice is the significantly increased mobility of the intrinsically flexible RBM (residues 470–490) in the SA55/SA58-RBD complex, which may allow for local changes in the RBM and modulation of the ACE2 binding affinity. While SA58 does not directly interfere with ACE2 and can engage both the up and down RBDs, SA55 only binds to the up RBD and can block ACE2 [57]. We found that the structural rigidification of the RBD regions via SA55/SA58 may be counteracted through elevated RBM mobility, which may lead RBM to adopt a variety of conformations that may not be fully compatible with ACE2 binding, The increased flexibility of the RBM region may allow for the hinge mechanism in which the rigid anchor residues 451–454 and 492–495 enable RBM movements that can expose and hide the ACE2 binding surface, as was also observed in other MD simulations [114,115].

Using this dynamic mechanism, SA55/SA58 binding may accomplish both effective neutralization via interaction with conserved epitopes critical for spike function and also control the ACE2 binding by imposing dynamic changes in the exposed RBM conformations of the RBD-up. Our dynamics analysis may be useful in light of recent proposals by Bloom and colleagues that mutations that move the RBD to more of an up conformation can generally increase the binding to ACE2 of the complete S protein but may also increase Ab neutralization since the RBD in the up conformation exposes more neutralizing epitopes (https://jbloomlab.org/posts/2024-11-20_escapecalc_update.html, (accessed on 19 December 2024)). They suggested that most mutations at sites 403, 405, 503, 504, and 505 may fall into this category as they affect RBD’s up–down movements; therefore, these mutations should be constrained in the virus evolution. According to our results, F3 Ab binding may induce the elevated RBM mobility in the RBD-up conformation, as well as modulation of the ACE2 exposure. At the same time, positions 503–505 are immobilized by SA55/SA58 binding and can form local hinges to control the RBD movements. Combined, these factors suggest that E1 and F3 Abs may also constrain the ability of the virus to evolve mutations without sacrificing the ACE2 binding or potentially exposing neutralizing epitopes. In this context, it may be worth noting that recent highly circulating variants KP.3.1.1 and XEC tend to escape neutralization via NTD mutations that induce the RBD-down form and block neutralization at the expense of efficient ACE2 binding [52,116].

### 3.4. Mutational Profiling of Protein Binding Interfaces with E1 and F3 Abs

**Figure 7:**
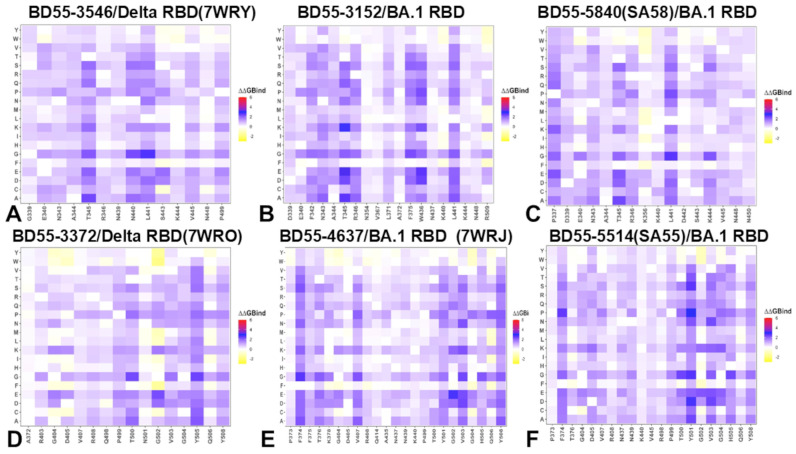
The ensemble-based mutational scanning of the binding of the SARS-CoV-2 S-RBD complexes with E1 and F3 Abs. The mutational scanning heatmaps of the RBD binding epitope residues in the S-RBD complexes with E1 Abs BD55-3546 (**A**), BD55-3152 (**B**), and SA58 (**C**), and S-RBD complexes with F3 group Abs BD55-3372 (**D**), BD55-4637 (**E**), and SA55 (**F**). The binding energy hotspots correspond to residues with high mutational sensitivity. The heatmaps show the computed binding free energy changes for 20 single mutations on the sites of variants. The squares on the heatmap are colored using a tricolored scale of blue, white, and yellow, with blue indicating the largest unfavorable effect on stability. The standard errors of the mean for the binding free energy changes were based on a different number of selected samples from a given trajectory (500 and 1000 samples) within 0.04-0.13 kcal/mol. The horizontal axis represents the RBD binding epitope residues. Binding epitope residues are the RBD binding interface residues that directly interact with antibodies. We used the BeAtMuSiC approach [101,102,103] contact predictor to identify the binding interface residues. Residues are considered part of the interface if they are within a defined cutoff distance (typically 5 Å) from atoms in the binding partner. The Y axis depicts all possible substitutions of a given RBD binding epitope residue, denoting mutations to letters using a single-letter annotation of the amino acid residues.

Using the conformational ensembles of the RBD complexes, we embarked on a structure-based mutational analysis of the S protein’s binding with Abs. To provide a systematic comparison, we constructed mutational heatmaps for the RBD interface residues of the S complexes with the E1 and F3 groups of Abs. We first analyzed the mutational heatmap for the E1 group. The strongest destabilization mutations for BD55-3546 included T345A (ΔΔG = 2.38 kcal/mol), T345G (ΔΔG = 2.16 kcal/mol), T345E (ΔΔG = 2.16 kcal/mol), T345S (ΔΔG = 1.91 kcal/mol), and N440A (ΔΔG =1.89 kcal/mol) (Figure 7A). T345 is surrounded by R105, W94, Y103, L96, Y91, and N92 Ab residues, establishing multiple favorable hydrogen bonding and van der Waals contacts. As a consequence, a mutational scanning identified multiple highly destabilizing substitutions (Figure 7A). BD55-3546 is also sensitive to mutations of N440, as N440 protrudes into a pocket formed by W50, N52, T55, I57, and T59 and forms extensive van der Waals and hydrogen-bond interactions (Figure 7A). These results are consistent with the escape profiles showing that BD55-3546 mainly escaped via T345 and N440 mutations [57]. These experimental studies also established that E1 Abs are predominantly susceptible to mutations of T345 and R346 residues [57]. Another E1 Ab BD55-3152 has binding energetic hotspots at T345, W436, L441, and 346 positions. For this Ab, the largest destabilization changes are associated with mutations of T345, including T345E (ΔΔG = 3.17 kcal/mol), T345K (ΔΔG = 2.82 kcal/mol), T345A (ΔΔG = 2.58 kcal/mol), and T345D (ΔΔG = 2.32 kcal/mol) (Figure 7B).

Hence, for both BD55-3546 and BD55-3152 Abs, T345 emerged as the dominant binding hotspot. As a result, the binding of these E1 Abs is highly sensitive and can be escaped via T345 mutations. Similarly, for SA58, the key binding hotspot is T345, where the T345A (ΔΔG = 2.36 kcal/mol), T345E (ΔΔG = 2.17 kcal/mol), and T345D (ΔΔG = 2.03 kcal/mol) mutations lead to large destabilizing changes (Figure 7C), which is consistent with experiments showing that mutations of T345 result in significant escape from SA58. In addition, we found that mutations of L441 may also reduce SA58 binding but the experiments showed only moderate sensitivity to mutations in this position. While T345 is clearly the dominant binding hotspot and a key target for escape mutations, it was concluded that T345 substitutions are unlikely to emerge as this site is critical to the proper glycosylation of N343, which is essential for RBD functioning [57].

The mutational heatmap for F3 BD55-3372 showed large destabilization free energies for mutations of Y505A (ΔΔG = 2.16 kcal/mol), Y505C (ΔΔG = 2.01 kcal/mol), and Y505D changes, as well as V503D (ΔΔG = 1.83 kcal/mol), Y508P (ΔΔG = 1.47 kcal/mol), G404E (ΔΔG = 1.67 kcal/mol), and G404K (ΔΔG = 1.52 kcal/mol) (Figure 7D). BD55-3372 is not susceptible to changes in Y508 but is sensitive to the changes in Y505, V503, and G504 (Figure 7D). Nevertheless, these sites are critical for ACE2 binding and both V503 and G504 are conserved, making it difficult for variants with mutations on these sites to emerge [57]. BD55-4637 has a rather unique escape profile, and is strongly affected by mutations in the 404, 503, 505, and 508 positions [57]. The mutational map identifies positions F374, V503, and Y508 as escape hotspots. Interestingly, mutations of V503 and Y508 are broadly destabilizing, with particularly unfavorable changes seen for V503D (ΔΔG = 2.38 kcal/mol), V503K (ΔΔG = 2.2 kcal/mol), V503N (ΔΔG = 2.24 kcal/mol), G504K (ΔΔG = 2.11 kcal/mol), Y508G (ΔΔG = 2.08 kcal/mol), and Y508N (ΔΔG = 1.97 kcal/mol) (Figure 7E). These results are consistent with the notion that BD55-4637 is sensitive to mutations of Y508 [57]. The detailed mutational heatmap of SA55’s interactions with the S protein showed large destabilization changes for Y501D (ΔΔG = 2.74 kcal/mol), Y501S (ΔΔG = 2.59 kcal/mol), V503D (ΔΔG = 2.41 kcal/mol), V503E (ΔΔG = 2.23 kcal/mol), and V503K mutations (ΔΔG = 2.02 kcal/mol) (Figure 7F). While these mutations are highly destabilizing for SA55 binding, these sites are fundamentally important for ACE2 binding and RBD functions. SA55 are sensitive to the changes in V503 and G504 but effective mutations for the destabilization of Ab binding are prohibitive as they would interfere with the key RBD functions [57]. As a result, evolution in these positions is highly constrained. A more detailed profiling of JN.1/KP.3 mutations against SA55 Ab showed only small destabilization changes upon mutations of T376A (ΔΔG = 0.81 kcal/mol), R403K (ΔΔG = 0.65 kcal/mol), D405N (ΔΔG = 0.79 kcal/mol), R408S (ΔΔG = 0.34 kcal/mol), L455S (ΔΔG = 0.7 kcal/mol), and F456L (ΔΔG = 0.51 kcal/mol) (Figure 7F). These changes reflect a mild loss in the RBD stability and binding interactions, which is consistent with the functional experiments [57] showing that group F3 Abs such as SA55 are not sensitive to the D405N and R408S mutations of BA.2, making SA55 effective against a broad spectrum of recent variants ranging from BA.2.86 to KP.2 and KP.3.

To summarize, the results of the mutational scanning using the rapid computation of binding free energy changes revealed binding hotspots for E1 and F3 Abs that are consistent with the experimental DMS data and immune escape centers. In particular, consistent with the experiments [57], mutational scanning predicted that the T345 and R346 residues would be dominant binding hotspots for the E1 group of Abs. Similarly, our results predicted that RBD sites V503, G504, and Y508 would be the key binding hotspots for the F3 group of Abs, consistent with experiments showing that these positions are major immune escape centers for these Abs.

### 3.5. MM-GBSA Analysis of the Binding Affinities

The experimental and computational studies suggested that the cross-neutralization of Ab activity against Omicron variants may be driven by the balance and tradeoff between multiple energetic factors and the interaction contributions of the evolving escape hotspots involved in antigenic drift and convergent evolution. However, the dynamic and energetic details quantifying the balance and contribution of these factors, particularly the balancing nature of specific interactions formed by Abs with the epitope hotspot residues, remain scarcely characterized. Here, using the conformational equilibrium ensembles and obtained MD simulations, we computed the binding free energies for the RBD-Ab complexes using the MM-GBSA method [104,105,106,107]. In this analysis, we focused on the binding free energy decomposition and an examination of the energetic contributions of individual RBD epitope residues. Through this analysis, we determined the binding hotspots for Ab binding and quantified the role of the van der Waals and electrostatic interactions in the binding mechanism. The computational predictions were systematically compared with the recent experimental data on average Ab escape scores (https://github.com/jbloomlab/SARS2-RBD-escape-calc/tree/main/Cao_data/JN1-evolving-Ab-response/data/DMS/Ab, (accessed on 19 December 2024)). These experimental data were generated with the escape calculator [117,118,119] and are reported in the updated analysis by Bloom’s lab (https://jbloomlab.github.io/SARS2-RBD-escape-calc/, (accessed on 19 December 2024)), which included the latest yeast-display DMS data obtained by Cao and colleagues [46]. In the MM-GBSA calculations, we examined whether the binding affinities and contributions of the major binding hotspots are largely determined by the van der Waals or electrostatic interactions and whether the immune escape positions are associated with the binding hotspots at which different energetic contributions act synergistically, leading to a significant loss of binding upon mutations.

**Figure 8:**
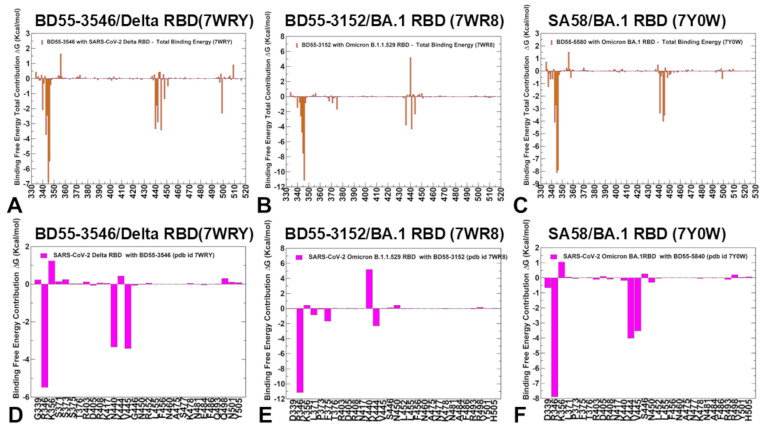
The residue-based profile of MM-GBSA binding energies and individual contributions. (**A**) The total MM-GBSA binding energy ΔG for the S-RBD complexes with E1 Abs BD55-3546 (**A**), BD55-3152 (**B**), and SA58 (**C**). A closeup of the total MM-GBSA binding energy ΔG per residue for the sites of JN.1 mutations in the S-RBD complexes with E1 Abs BD55-3546 (**D**), BD55-3152 (**E**), and SA58 (**F**). The MM-GBSA contributions were evaluated using 1000 samples from the equilibrium MD simulations of respective RBD-ACE2 complexes. The statistical errors were estimated on the basis of the deviation from the block average and are within 0.18–0.32 kcal/mol.

We started with an MM-GBSA analysis of the E1 group Abs for which the residue decomposition of the total energy revealed strong and consistent binding hotspots for the T345 and R346 positions (Figure 8A–C). For the BD55-3546 binding, the total binding energies in these positions amounted to ΔG = −6.88 kcal/mol and ΔG = −5.48 kcal/mol (Figure 8A,D). Similar deep peaks in the binding energies for the T345/R346 positions are seen for the BD55-3152 (ΔG = −7.53 kcal/mol and ΔG = −11.13 kcal/mol) (Figure 8B,E) and SA58 binding (ΔG = −8.10 kcal/mol and ΔG = −7.89 kcal/mol) (Figure 8C,F). Notably, the residue decomposition also showed very favorable binding for adjacent positions N343 and A344 (Figure 8). The epitopes of these E1 Abs include the N343 glycan, which plays a critical role in modulating the RBD conformation [57]. We also observed strong binding contributions of E340, L441, and V345 for BD55-3546 binding (Figure 8A,D) and corresponding significant binding components of L441, K444, and V445 for SA58 (Figure 8C,F), while for BD55-3152, strong binding contributions were seen for L441 and K444 (Figure 8B,E). Interestingly, for SA58 Ab, the tiers containing the strongest binding hotspots included T345/R346 in the first tier and N343, L441, K444, and V445 in the second tier (Figure 8C,F). We suggested that the positions of the strongest binding hotspots should correspond to the major Ab escape positions.

By comparing the predicted residue-based binding free energy contributions with the averaged Ab escape scores (Supporting Information, Figure S3), we found an excellent overall agreement between the predicted and experimental data. Indeed, as predicted, the strongest immune escape sites for E1 Abs were T345 and R346 (Figure 8, Supporting Information, Figure S3). In particular, for BD55-3546, the main escape positions were E340, T345, R346 and L441, as was predicted in the energy analysis. For BD55-3142, the experiments revealed T345, R346, and L441 to be major escape hotspots, which is also consistent with our predictions. Finally, for SA58, the experiments singled out E340, T345, R346, L441, K444, and L452 as the dominant escape hotspots based on the neutralization data against the JN.1 and JN.1 subvariants (https://jbloomlab.github.io/SARS2-RBD-escape-calc/, (accessed on 19 December 2024)).

According to the experimental data, neutralization by SA58 is moderately affected by T345N, R346Q, and R346T, while it is strongly reduced by E340D and escaped by the E340K and K444E mutations [57]. According to our computations, the top binding hotspots are E340, T345, R346, N342, K444, V445, and L441 (Figure 8C,F), showing the predictive ability of binding computations when identifying key binding hotspots and associating the strongest binding centers with major escape positions. Combined with the accompanied mutational scanning of the RBD epitope residues, this strategy can represent a robust way to suggest escape sites and important escape mutations.

**Figure 9:**
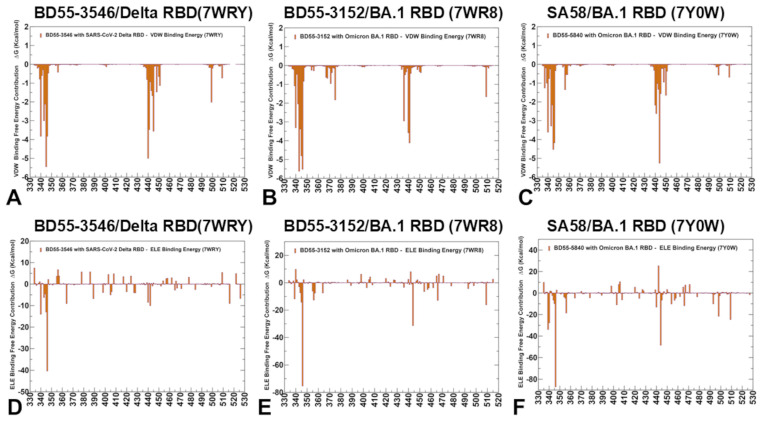
The residue-based profiles the MM-GBSA binding energy contributions. (**A**) The van der Waals contribution to the total MM-GBSA binding energy for the S-RBD complexes with E1 Abs BD55-3546 (**A**), BD55-3152 (**B**), and SA58 (**C**). The electrostatic contribution to the total MM-GBSA binding energy for the S-RBD complexes the S-RBD complexes with E1 Abs BD55-3546 (**D**), BD55-3152 (**E**), and SA58 (**F**). The MM-GBSA contributions are evaluated using 1000 samples from the equilibrium MD simulations of the respective RBD-ACE2 complexes.

We also performed a contribution-based energetic analysis (Figure 9) for the E1 group of Abs, showing the strong and synergistic contributions of both van der Waals and electrostatic components of the key hotspots T345 and K346. While the electrostatic contributions are often offset by the corresponding desolvation values (Figure 9, Tables S1–S3), for T345 and R346, the electrostatic interactions are strong drivers of binding and can only partly be counteracted by the solvation penalties. In general, our results indicated that the dominant binding hotspots T345 and K346 for E1 Abs, and their respective role as major Ab escape positions, are determined through the synergistic hydrophobic and electrostatic binding interactions of these residues (Figure 9). For BD55-3152, in addition to the T345 and R346 hotspots, we found strong electrostatic interactions with K444, and the van der Waals component for this residue was rather small (Figure 9B,E). For this complex, E340 emerged as a moderately favorable site but the electrostatic interactions between E340 and BD55-3152 were highly unfavorable (Figure 9B,E).

The synergistic contribution of both electrostatic and hydrophobic interactions between K444 and SA58 resulted in this position being predicted to be a strong binding hotspot, along with T345 and R346 (Figure 9C,F). As a result, most mutations in these positions can induce significant binding loss, leading to the emergence of escape mechanisms through convenient-for-virus modifications that do not compromise ACE2 binding. Indeed, SA58 showed a decrease in activity due to K444N and completely escaped through the combination of K444N and T345P mutations in the experimental assays [57]. Mutations at these positions can disrupt these interactions, reducing the binding affinity of the Abs. In addition, T345 and K346 are located in regions of RBD that are crucial for maintaining its structural integrity, and changes in these residues can alter the conformation of the RBD, making it more difficult for Abs to bind effectively. Interestingly, for the second tier of binding hotspots, including N343, L441, K444, and V445 residues, the total binding energy is mainly determined by strong van der Waals and hydrophobic interactions while the electrostatic interactions are mostly compensated by the corresponding unfavorable desolvation’s contribution (Figure 9). The important result of this analysis is that MM-GBSA computations, combined with mutational scanning, can efficiently predict the binding hotspots and corresponding immune escape positions. Secondly, for E1 group Abs, the dominant role of the T345 and K346 hotspots and immune escape centers may be due to the synergistic contribution of both van der Waals and electrostatic interactions. The results also revealed that the binding of the E1 group Abs is determined by a small number of key binding energy hotspots, T345, R346, and K444, while the contribution of other binding epitope positions is fairly small (Figure 8 and Figure 9). Moreover, we noticed that the long-range electrostatic interactions between by the E1 group Abs were generally moderate and often unfavorable, which is consistent with the notion that the accumulation of positive charges on the RBD can often mediate immune resistance via positively charged S RBD-recognition surfaces [74,75,76]. Strikingly, however, the complementary electrostatic interactions of the E1 group Abs with T345, R346, and K444 are the main determinants of the binding and the narrow scope of immune escape mutations (Figure 8 and Figure 9).These results agree with the experiments showing that T345, K346, and K444 are involved in electrostatic interactions that are crucial for the binding of E1 Abs to the RBD [57].

**Figure 10:**
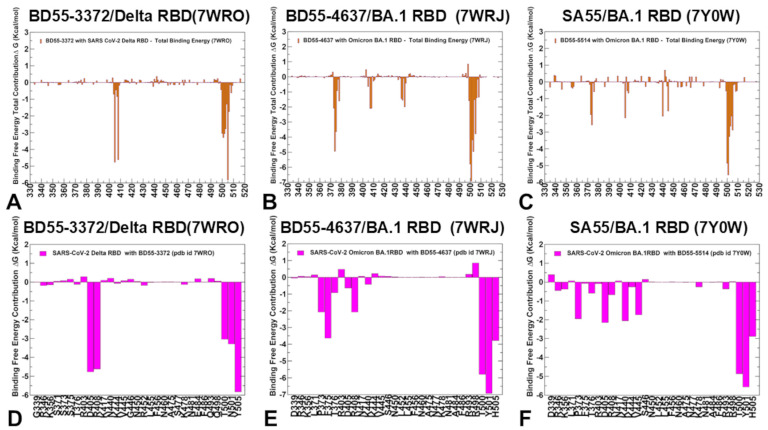
The residue-based profile of the MM-GBSA binding energies. (**A**) The total MM-GBSA binding energy ΔG’s contribution to the S-RBD complexes with F3 group Abs BD55-3372 (A), BD55-4637 (**B**), and SA55 (**C**). A closeup of the total MM-GBSA binding energy ΔG per residue for the sites of JN.1 mutations in the S-RBD complexes with F3 group Abs BD55-3372 (**D**), BD55-4637 (**E**), and SA55 (**F**). The MM-GBSA contributions are evaluated using 1000 samples from the equilibrium MD simulations of respective RBD-ACE2 complexes. The statistical errors were estimated on the basis of the deviation from the block average and are within 0.16–0.45 kcal/mol.

The energetic drivers of the F3 group of Abs are largely determined by the contributions of the RBD residues 500–508, which are also critical for RBD stability and ACE2 binding (Figure 10). For BD55-3372, the top binding hotspots are Y505, D405, and R408 (ΔG = −5.82 kcal/mol, ΔG = −4.76 kcal/mol, and ΔG = −4.76 kcal/mol, respectively) (Figure 10A,D). Only slightly less dominant are positions 501, 502, and 503. The experiment-based Ab escape scores favored V503, G504, and D405 as the main escape positions (Supporting Information, Figure S3), mostly because mutations in T500 and Y501 cannot emerge due to functional constraints, even though mutations in these positions could significantly diminish BD55-3372 binding (Figure 10). Y508 is also essential to ACE2 binding and is less able to evolve [57]. BD55-3372 is sensitive to mutations of V503 and G504 that are less critical for ACE2 binding but are still constrained for evolution due to the role of these sites in critical RBD functions.

For BD55-4637, the energy contributions revealed major binding hotspots in positions Y501, T500, V503, F374, G502, and H505 (Figure 10B,E). Importantly, these sites, and particularly Y501 and T500, are fundamental to the ACE2 binding function, while F374, G502, and V503 are also involved in the RBD stability and ACE2 interactions. The second tier of binding hotspots included F375, R408, N439, and K478 (Figure 10B,E). The experiments showed sites Y508, G502, V503, N439, and F378 to be major escape centers (Supporting Information, Figure S3), thus revealing good general agreement with the binding energy computations. Similarly, for SA55, our result predicted the Y501, T500, G502, H505, V503, G504, F374, D405, K440, and V445 positions to be important RBD positions for binding, showing an expanded set of binding hotspots but still predicting regions 500–505 to be the dominant regions for binding (Figure 10C,F). The positions of immune escape for SA55 are G502, H505, V503, G504, and Y508 (Supporting Information, Figure S3). The efficacy of SA55 is affected by Y508H, moderately affected by G504S, and strongly affected by K440E, while V503E and G504D allow for escape [57]. Hence, our computations correctly predict the most critical escape centers for SA55.

**Figure 11:**
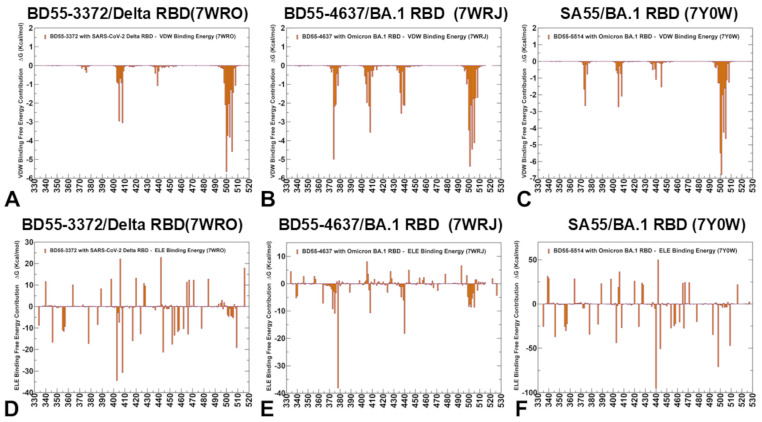
The residue-based profiles of the MM-GBSA binding energy contributions. (**A**) The van der Waals contribution to the total MM-GBSA binding energy for the S-RBD complexes with F3 group Abs BD55-3372 (**A**), BD55-4637 (**B**), and SA55 (**C**). The electrostatic contribution to the total MM-GBSA binding energy for the S-RBD complexes with F3 group Abs BD55-3372 (**D**), BD55-4637 (**E**), and SA55 (**F**).

We followed with an analysis of the individual energetic contributions of the F3 group Abs, revealing the major role of the van der Waals interaction component (Figure 11). This is consistent with the hydrophobic nature of the critical binding hotspot region (residues 500–505), which has the same overall shape and the same relative residue contributions to the total energy (Figure 10) and the van der Waals interaction component (Figure 11). For BD55-3372, the favorable electrostatic interactions are formed with the R403, R408, and K444 sites but these contributions are often offset by desolvation penalties, with the exception of R408 (Figure 11D).

For BD55-4637, the electrostatics drives the binding energy for K378 and R408 (Figure 11E). For SA55, the electrostatic component is the dominant force behind the energetics of the binding hotspots K378, K440, R403, R408, and K444 (Figure 11F). For these F3 Abs, the electrostatic interactions between the RBD residues and F3 Abs include both favorable and highly unfavorable contributions (Figure 11D–F). For these Abs, we also observed that some secondary tier hotspots emerge when both van der Waals, and electrostatic contributions act synergistically to mediate the strong binding. Indeed, for BD55-3372, the favorable electrostatic contributions of R403 and R408 are synergistic with the favorable hydrophobic contributions and van der Waals contributions (Figure 11A,D). The strong electrostatic interactions with K378 in the complex with BD55-4637 (Figure 11E) and with K440 in the complex with SA55 (Figure 11F) are the determining factors for these hotspots, which is consistent with the experiments showing that the SA55 binding can be significantly compromised by changes in the charge caused by the K440E mutation [57]. The K378 residue is less commonly targeted by specific Abs, but it can be involved in the binding sites of broadly neutralizing Abs that recognize conserved regions of the RBD. K444 is more frequently targeted by neutralizing Abs, including group D Abs, such as REGN-10987, LY-CoV1404, and COV2-2130, that bind to the linear epitope 440–449 and showed effectiveness in neutralizing various Omicron variants [54,55,56,57,58]. N440K mutants, along with K444N/T, were resistant to C135 Ab, which is a class 3 Ab targeting the same binding epitope as the F3 group Abs [120].

To summarize the key findings of this analysis, the MM-GBSA analysis identified T345 and R346 as the dominant binding hotspots for the E1 group of antibodies (BD55-3546, BD55-3152, and SA58). These residues also correspond to major immune escape positions, as confirmed by the experimental escape scores. For the F3 group of antibodies (BD55-3372, BD55-4637, and SA55), the binding hotspots are concentrated in the RBD region spanning residues 500–508, which are critical for both RBD stability and ACE2 binding. Key residues such as Y501, T500, and V503 are identified as major binding and escape sites. We also found that the binding affinities of the E1 group antibodies are driven by a combination of van der Waals and electrostatic interactions, with T345 and R346 showing strong electrostatic contributions that are only partially offset by the desolvation penalties. For the F3 group antibodies, van der Waals interactions dominate the binding energetics, particularly in the hydrophobic region of residues 500–505. However, electrostatic interactions play a significant role driving the binding contribution of RBD residues K378, K440, and R408.

An important finding of this analysis is that the synergistic contributions of the van der Waals and electrostatic interactions at key hotspots (e.g., T345, R346, and K444) are crucial for antibody binding. Mutations at these positions disrupt these interactions, leading to significant binding loss and immune escape. The findings suggest that SARS-CoV-2 variants evolve mutations that balance antibody escape with maintained or enhanced ACE2 affinity. This dual evolutionary pressure is critical for viral fitness and immune evasion.

## 4. Discussion

The SARS-CoV-2 virus must maintain a delicate balance between binding effectively to the ACE2 receptor and evading Abs. Broadly neutralizing monoclonal Abs recognized the conserved antigenic sites outside the RBM as sites of Omicron-mediated immune evasion, marking a significant antigenic shift in SARS-CoV-2 [46,121]. The recent evidence indicated that the latest variants and JN.1 sublineages can display lower affinity to ACE2 and the enhanced antibody evasion profile [46,121]. Mutations that shift the electrostatic potential can help the virus achieve this balance between ACE2 binding and immune escape. In general, the emergence of the JN.1 descendants, accompanied by the increased Ab evasion and decrease in ACE2 affinity, suggest the evolutionary objectives of the virus. In this context, it is important to understand the energetic mechanisms underlying the evolving Ab response to Omicron’s antigenic shift to JN.1, through which F3 Abs retain potency and neutralizing activity against JN.1 subvariants, whereas Abs from other groups largely escape [46,54,55,56,57,58]. Mutational scanning and binding free energy calculations provide insights into why E1 and F3 Abs targeting more conserved epitope surfaces, particularly F3, are effective and exhibit broadly neutralizing activity against the latest variants. The results showed that binding E1 Abs is strongly susceptible to changes in charges and the electrostatic interactions between T345 and R346, which are the dominant binding hotspots and immune escape centers. As a result, mutations R346Q and R346T emerged as important changes regarding Ab escape for this group [57]. On the other hand, binding F3 Abs is driven primarily by the hydrophobic interactions that are formed with the RBD residues 500–508, which are implicated in stability, ACE2 binding, and critical spike functions. Our results provide an interesting rationale for the experimental observation that mutations in the epitope consisting of sites R403, D405, V503, G504, and H505 can allow for significant escape from binding by neutralizing Abs targeting the RBD although these mutations have not emerged in the circulation [46,56,58]. We suggest that the evolution of the RBD mutations in these positions may be constrained due to synergistic energetic couplings between T500/Y501, which are indispensable for ACE2 binding and structurally proximal V503, G504, and H505 sites.

According to our results, the binding energetics for the E1 and F3 groups of Abs are driven by a small number of key binding hotspots that can also include positively charged residues R346, K378, R403, R408, K440, and K444, forming strong electrostatic interactions. In particular, for F3 Abs, we found that the electrostatic interactions are determining factors for the binding hotspots K378, K440, R403, R408, and K444. We previously showed that mutational sites that contribute to the ACE2 binding affinity can include RBD residues K378, R403, K424, K440, K444, K460, N477, and K478 owing to electrostatic interactions mediated by lysine residues [64,66,81]. Concurrently, the results of this study suggested that the F3 group Abs may achieve broad neutralizing activity against JN.1 variants by targeting the specific charged RBD sites that could allow for strong Ab binding but are also important for ACE2 binding. The observed immune escape mutations in these positions, K444T/N and K440T/E, are detrimental to Ab binding [57] as they reduce the positive electrostatic potential of the RBD and possibly the conformation of the RBD, which may also adversely affect RBD’s binding to ACE2. Another functional study highlighted the expected disruptions to the electrostatic interactions with the Abs resulting from the K444T and the R346T RBD mutations that can boost the immune escape profile [122]. Based on our analysis, we argue that the broadly neutralizing activity of E1 and F3 Abs against the latest variants may also be partly attributed to the narrowed repertoire of immune escape mutations on the RBD to combat the binding of these Abs, where some of these mutations are forced to reduce the positive charge of the RBD.

A phylogenetic analysis of the latest SARS-CoV-2 Omicron clades (JN.1, KP.2, and KP.3) revealed several recurrent mutations at key hotspots (T345, R346, L455, and F456) that are consistent with the mutational scanning and MM-GBSA binding results presented in this study. Notably, mutations such as R346T, L455F, and F456L were observed across these clades and have been shown to play a critical role in modulating antibody binding and ACE2 affinity. These mutations represent key evolutionary adaptations that align with the functional hotspots identified in our binding studies. For example, R346T, which is prevalent in KP.2 and KP.3, reduces binding to SA55 and S58 antibodies while maintaining ACE2 affinity, as demonstrated by our MM-GBSA binding studies. These findings highlight the functional significance of convergent evolution in shaping the binding properties of the SARS-CoV-2 spike protein. These mutations often arise in response to immune pressure, leading to the evolution of viral variants that can evade antibody neutralization while maintaining the ACE2 binding affinity. The MM-GBSA predictions complement this by quantifying the energetic contributions of these mutations to antibody binding, offering a mechanistic explanation for the observed evolutionary trends.

It is also worth noting that although the NTD mutational change makes the charge more negative [123], the overall charge in the S protein is increasing. However, recently emerged lineages and escape mutants showed greater diversity in the composition of ionizable amino acids. A recent analysis found that the progressive increase in the positive charge of the S protein may have reached a peak with XBB lineages; the total charge of the S protein was still positive but showed greater variability. However, JN.1 and its subvariants still displayed an increasing number of positively charged residues. [124]. Our analysis suggested that these positively charged positions in JN.1 subvariants can be successfully exploited by E1 and F3 Abs, forcing the virus to alter the charge of the escape centers. According to our results, the E1 and F3 groups of Abs effectively exploit binding hotspot clusters of hydrophobic sites that are critical for RBD functions, along with the selective complementary targeting of positively charged RBD sites that are important for ACE2 binding. Together with targeting conserved epitopes, these groups of Abs can expand the breadth of neutralization and increase the resilience to antigenic shifts associated with viral evolution. These findings suggest that RBD mutations and their associated immune escape may reach a plateau under the constraints of RBD’s stability and ACE2 binding and that selective pressures could be used to explore epistatic effects and localized electrostatic changes that may boost immune evasion.

While our study provides a detailed mechanistic understanding of Ab-RBD interactions and predicts the key escape mutations, a full assessment of the escape potential of these mutations requires more extensive and complete experimental validation. To address this, we compared our computational predictions with all available experimental data, such as the reduced binding affinity of SA55 due to K440E and V503E mutations and the escape of SA58 via K444N and T345P mutations. These comparisons demonstrate the reliability of our approach and highlight the importance of integrating computational and experimental methods to fully evaluate immune escape. Future studies could expand on this work by incorporating experimental data from neutralization assays, deep mutational scanning, or structural studies to validate and refine our predictions. Such an integrated approach would provide a more comprehensive understanding of viral evolution and inform the development of effective therapeutic strategies.

While our computational approach provides valuable insights into the antibody escape mechanisms, it is important to acknowledge its limitations. For instance, the MM/GBSA method used to calculate binding energies does not explicitly account for entropic contributions and relies on implicit solvent models to account for the ionic effects. These simplifications are necessary to achieve computational efficiency but may affect the absolute accuracy of the binding energy predictions. However, our focus on relative binding energy changes (ΔΔG) mitigates some of these limitations, and the qualitative agreement with the available experimental data (Figure S3) supports the robustness of our predictions. Additionally, while this study focuses on the RBD-Ab interactions, RBD-ACE2 binding is another critical balancing factor influencing viral fitness and immune evasion. Our previous work extensively characterized RBD-ACE2 binding for key variants, including JN.1, KP.2, and KP.3, revealing that many mutations balance antibody escape with maintained or enhanced ACE2 affinity [62,66]. Integrating RBD-ACE2 binding data with antibody escape analyses provides a more comprehensive understanding of viral evolution. The findings presented in this study, combined with our previous work on RBD-ACE2 binding [66], enabled a quantitative analysis of the major thermodynamic drivers of viral evolution and accurate identification of the binding energy hotspots and escape centers in the S-RBD protein, revealing a complex and evolving interplay between immune evasion and receptor binding.

## 5. Conclusions

Using dynamic ensembles of the S-Ab complexes and systematic mutational scanning of the RBD residues that bind with Abs, we characterized patterns of mutational sensitivity and created mutational scanning heatmaps to identify escape hotspots for a panel of E1 and F3 Abs. Mutational scanning using the rapid computation of binding free energy changes revealed binding hotspots for E1 and F3 Abs that are consistent with the experimental DMS data and immune escape centers. In particular, consistent with the experiments [57], mutational scanning predicted T345 and R346 residues to be the dominant binding hotspots for the E1 group of Abs. Similarly, our results predicted RBD sites V503, G504, and Y508 to be the key binding hotspots for the F3 group of Abs, which agrees with the experiments showing that these positions are major immune escape centers for these Abs. MM-GBSA binding free energy computations revealed group-specific binding energy hotspots that are consistent with the experimentally determined immune escape centers. Our analysis suggested that the E1 and F3 groups of Abs targeting binding epitopes may exploit strong hydrophobic interactions with the binding epitope hotspots that are critical for RBD stability and ACE2 binding, while escape mutations tend to emerge in sites associated with synergistically strong van der Waals and electrostatic interactions. Our analysis shows that the emergence of a small number of immune escape positions for the E1 group Abs are associated with the R346 and K444 positions. The binding of F3 group Abs yields an Ab-specific balance between the van der Waals contributions and electrostatic contributions. Our analysis confirms that V503, G504, and H505 correspond to the key escape positions for F3 Abs, as T500 and Y501 are critical for ACE2’s binding function. The second strongest group of binding hotspots for F3 Abs is R403 and D405, in which mutations are typically tolerant of the ACE2 affinity. In addition, despite the common binding epitope, different F3 Abs can also have unique binding hotspots in RBD positions F374, T376, and K378. These results are consistent with the experimental data on the strongest RBD sites of immune escape from binding by neutralizing Abs.

Recent studies of emerging Omicron variants suggested that the evolutionary paths for significant improvements in the binding affinity of the Omicron RBD variants with ACE2 are relatively narrow and may involve the use of convergent mutational hotspots to optimize immune escape while retaining sufficient ACE2 affinity. These mechanisms, based on convergent adaptation, may determine the scope of “evolutionary opportunities” for the virus to establish new mutations that improve immune resistance without compromising ACE2 binding affinity and stability. Our results may be helpful in rationalizing the roles and synergistic contributions of the hydrophobic and electrostatic drivers of binding for broadly neutralizing groups of Abs by exploiting their electrostatic complementarity to a targeted set of positively charged RBD residues and binding to hydrophobic patches of functionally important RBD positions on conserved epitopes.

## Data Availability

The original contributions presented in this study are included in the article/Supplementary Materials. Crystal structures were obtained and downloaded from the Protein Data Bank (http://www.rcsb.org, (accessed on 19 December 2024)). All simulations were performed using NAMD 2.13 package that was obtained from website https://www.ks.uiuc.edu/Development/Download/, (accessed on 19 December 2024). All simulations were performed using the all-atom additive CHARMM36 protein force field, which can be obtained from http://mackerell.umaryland.edu/charmm_ff.shtml, (accessed on 19 December 2024). The rendering of protein structures was achieved with the interactive visualization program UCSF ChimeraX package (https://www.rbvi.ucsf.edu/chimerax/, (accessed on 19 December 2024)) and Pymol (https://pymol.org/2/, (accessed on 19 December 2024)).

## Supplementary Materials

The following supporting information can be downloaded at: https://www.mdpi.com/article/10.3390/biom15020249/s1, Figure S1 highlights the evolution of SARSB-CoV-2 lineages. Figure S2 represents the prevalence of the JN.1 variant among all the samples tested worldwide. Figure S3 presents the residue-based averaged Ab escape scores, derived from the latest experimental data [46]. Table S1 lists the Omicron mutations in the S protein for a variety of variants, including JN.1, KP.2, and KP.3. Table S2 lists the binding epitope residues and contacts with the RBD for BD55-3546. Table S3 lists the binding epitope residues and contacts with the RBD for BD55-3152. Table S4 lists the binding epitope residues and contacts with the RBD for SA58. Table S5 lists the binding epitope residues and contacts with the RBD for BD55-3372. Table S6 lists the binding epitope residues and contacts with the RBD for BD55-4637. Table S7 lists the binding epitope residues and contacts with the RBD for SA55.
