# Supplementary material for: Quantitative Characterization and Prediction of the Binding Determinants and Immune Escape Hotspots for Groups of Broadly Neutralizing Antibodies Against Omicron Variants: Atomistic Modeling of the SARS-CoV-2 Spike Complexes with Antibodies
Source: Biomolecules. 2025 Feb 8;15(2):249. doi: 10.3390/biom15020249 (PMC11853647; doi:10.3390/biom15020249)
Supplement: Supplementary file 1 [file biomolecules-15-00249-s001.zip › biomolecules-3410750-supplementary.pdf]

Supplementary Materials

Quantitative Characterization and Prediction of the Binding Determinants and Immune Escape Hotspots for Groups of Broadly Neutralizing Antibodies Against Omicron Variants: Atomistic Modeling of the SARS-CoV-2 Spike Complexes with Antibodies

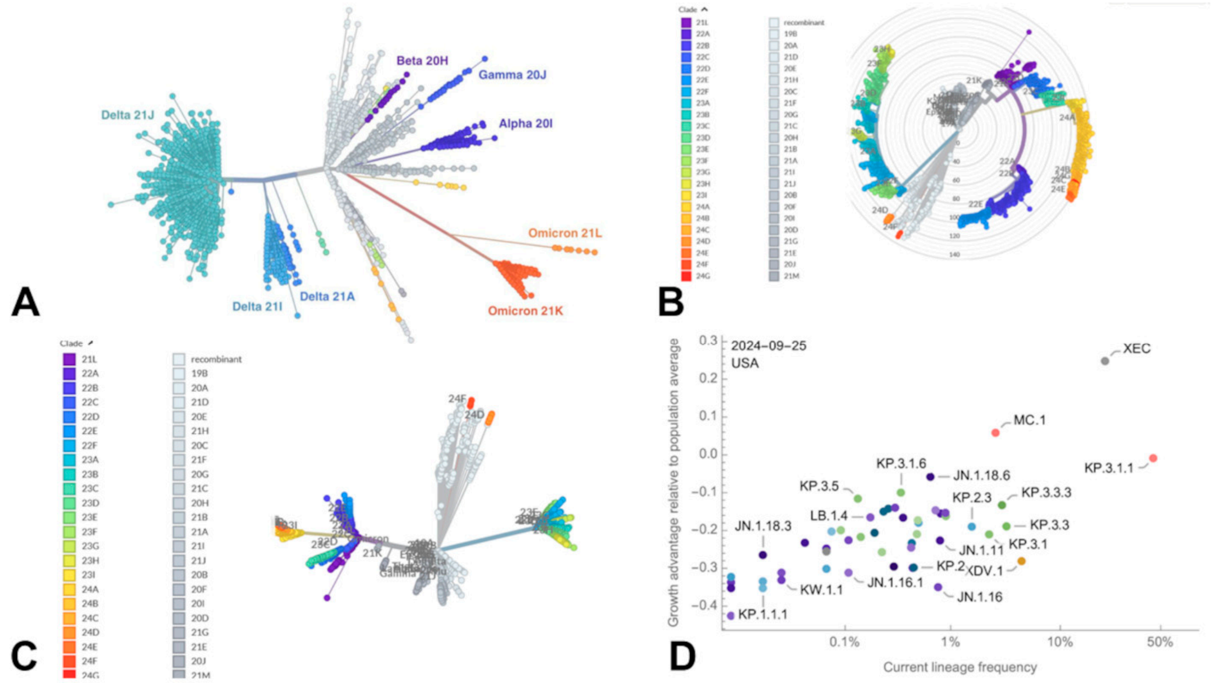

Figure S1. An overview of the phylogenetic analysis and SARS-CoV-2 clade classification highlights the evolution of SARSB-CoV-2 lineages. A comparison of evolutionary divergence between Omicron variants. Alpha, Beta and Gamma are each about as divergent from each other in terms of amino acid changes across the genome as Omicron 21K and 21L are from each other (A), radial phylogenetic tree representation (B) and unrooted phylogenetic tree representation (C). The recent data on growth advantage relative to population average in US (https://github.com/nextstrain/ncov/pull/1152) (D). These plots illustrate evolutionary trajectories of Omicron lineages can proceed through complex recombination, antigenic drift and convergent evolution. The graphs are generated using Nextstrain, an open-source project for real time tracking of evolving pathogen populations (https://nextstrain.org/).

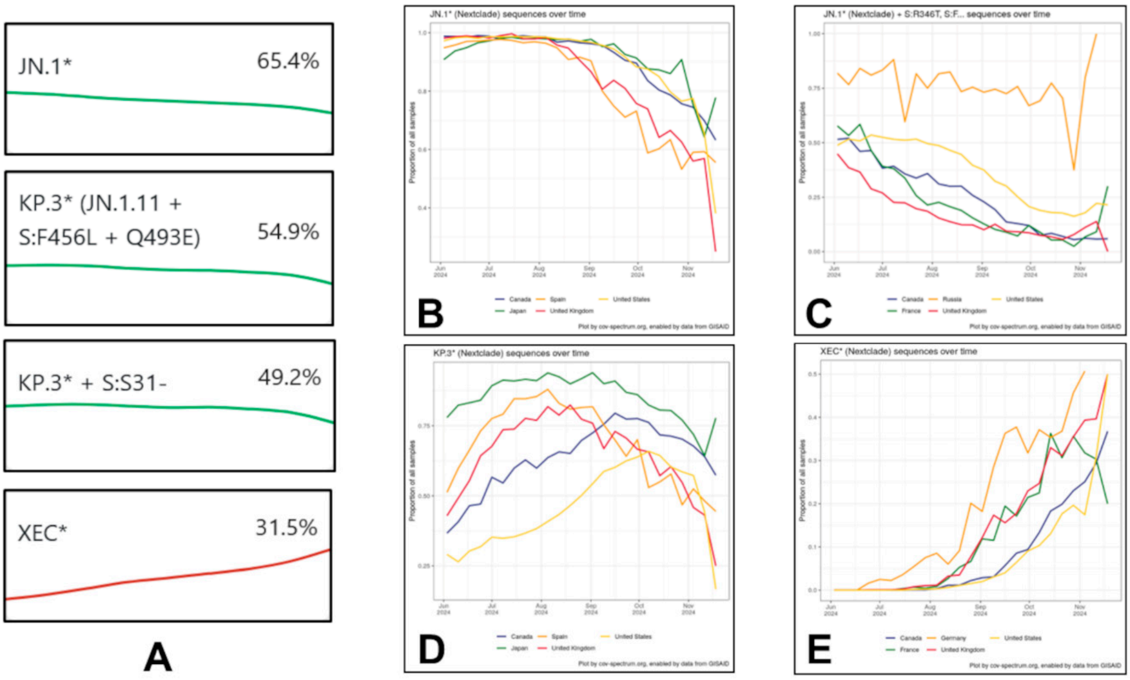

Figure S2. Cov-Spectrum data (https://cov-spectrum.org/explore/World/AllSamples/) for the dominant variants of the samples collected and analyzed within the specified date range (June 3, 2024, to November 26, 2024). This percentage represents the prevalence of the JN.1 variant among all the samples in the world tested during that period. The high proportion of the world samples collected and analyzed during this latest period is dominated by other JN.1 descendants, particularly 54.8% for KP.3, 49.2 % for KP.3.1.1 and 31.5 % for XEC variant. Notice that the sum of percentages for the presented variants on Cov-Spectrum can exceed 100% because some samples may contain multiple variants

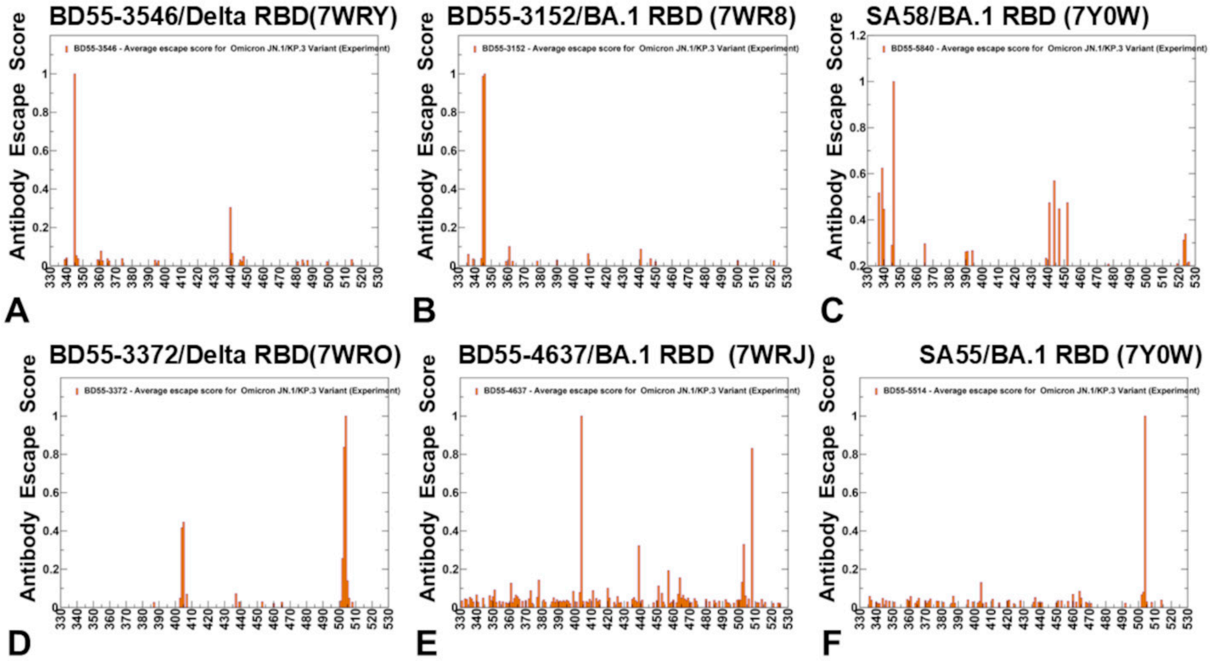

Figure S3. The residue-based averaged Ab escape scores derived from the latest experimental data [46] for E1 group Abs BD55-3546 (A), BD55-3152 (B), SA58 (C), and F3 group Ab BD55-3372 (D), BD55-4637 (E) and SA55 (F).

Table S1. Mutational landscape of the Omicron variants.

Variant	Mutational landscape

BA.1	A67, T95I, G339D, S371L, S373P, S375F, K417N, N440K, G446S, S477N, T478K, E484A, Q493R, G496S, Q498R, N501Y, Y505H, T547K, D614G, H655Y, N679K, P681H, N764K, D796Y, N856K, Q954H, N969K, L981F
BA.2	T19I, G142D, V213G, G339D, S371F, S373P, S375F, T376A, D405N, R408S, K417N, N440K, S477N, T478K, E484A, Q493R, Q498R, N501Y, Y505H, D614G, H655Y, N679K, P681H, N764K, D796Y, Q954H, N969K
XBB.1.5	T19I, V83A, G142D, Del144, H146Q, Q183E, V213E, G252V, G339H, R346T, L368I, S371F, S373P, S375F, T376A, D405N, R408S, K417N, N440K, V445P, G446S,N460K, S477N, T478K, E484A, F486P, F490S, R493Q reversal, Q498R, N501Y, Y505H, D614G, H655Y, N679K, P681H, N764K, D796Y, Q954H, N969K
BA.2.86	T19I, R21T, S50L, del69-70,V127F, delY144, F157S, R158G, delN211, L213I, L226F, H25N,A264D, I332V, D339H, K356T, R403K, V445H, G446S, N450D, L452W, N460K, N481K, del V483, A484K, F486P, R493Q, E554K, A570V, P612S, I670V, H68R, D939F, P1143L
JN.1	T19I, R21T, S50L, del69-70,V127F, delY144, F157S, R158G, delN211, L213I, L226F, H25N, A264D, I332V, D339H, K356T, R403K, V445H, G446S, N450D, L452W, **L455S,** N460K, N481K, del V483, A484K, F486P, R493Q, E554K, A570V, P612S, I670V, H68R, D939F, P1143L
KP.2	**JN.1 + S:R346T, S:F456L, S:V1104L**<br>T19I, R21T, S50L, del69-70,V127F, delY144, F157S, R158G, delN211, L213I, L226F, H25N,A264D, I332V, D339H, **R346T**, K356T, R403K, V445H, G446S, N450D, L452W, **L455S, F456L**, N460K, N481K, del V483, A484K, F486P, R493Q, E554K, A570V, P612S, I670V, H68R, D939F, **V1104L,** P1143L
KP.3	**JN.1 + S:F456L, S:Q493E, S:V1104L**<br>T19I, R21T, S50L, del69-70,V127F, delY144, F157S, R158G, delN211, L213I, L226F, H25N,A264D, I332V, D339H, K356T, R403K, V445H, G446, N450D, L452W, **L455S, F456L**, N460K, N481K, del V483, A484K, F486P, **Q493E**, E554K, A570V, P612S, I670V, H68R, D939F, **V1104L,** P1143L
LB.1	**JN.1+ S:S31-, S:Q183H, S:R346T, S:F456L**<br>T19I, R21T, **S31-**, S50L, del69-70,V127F, delY144, F157S, R158G, **Q183H**, delN211, L213I, L226F, H25N,A264D, I332V, D339H, **R346T,** K356T, R403K, V445H, G446S, N450D, L452W, **L455S, F456L**, N460K, N481K, del V483, A484K, F486P, R493Q, E554K, A570V, P612S, I670V, H68R, D939F, P1143L
XEC	**JN.1 + S:T22N, S:F59S, S:F456L, S:Q493E, S:V1104L**<br>T19I, R21T, **T22N**, S50L, **F59S**, del69-70,V127F, delY144, F157S, R158G, delN211, L213I, L226F, H25N,A264D, I332V, D339H, K356T, R403K, V445H, G446S, N450D, L452W, **L455S,** F456L. N460K, N481K, del V483, A484K, F486P, **Q493E**, E554K, A570V, P612S, I670V, H68R, D939F, **V1104L**, P1143L

Table S2. The list of the binding epitope residues and BD55-3546 Ab residues forming interfacial contacts

RBD Residue	RBD Residue Number	RBD Chain	Antibody residue	Antibody residue number	Antibody chain
GLY	339	R	TYR	93	L
GLY	339	R	TRP	94	L
GLU	340	R	ASN	28	L
GLU	340	R	TYR	93	L
GLU	340	R	HIS	27	L
VAL	341	R	TYR	93	L
ASN	343	R	TYR	93	L
ASN	343	R	PRO	95	L
ASN	343	R	ASN	92	L
ASN	343	R	TRP	94	L
ALA	344	R	TRP	94	L
ALA	344	R	TYR	93	L
ALA	344	R	ASN	92	L
THR	345	R	LEU	102	H
THR	345	R	TYR	93	L
THR	345	R	ARG	105	H
THR	345	R	TRP	94	L
THR	345	R	TYR	103	H
THR	345	R	LEU	96	L
THR	345	R	TYR	91	L
THR	345	R	ASN	92	L
ARG	346	R	TYR	103	H
ARG	346	R	GLU	104	H
ARG	346	R	ASN	92	L
PHE	347	R	TYR	103	H
LYS	356	R	TYR	93	L
ASN	439	R	ASN	52	H
ASN	439	R	THR	55	H
ASN	440	R	PRO	58	H
ASN	440	R	ILE	57	H
ASN	440	R	THR	59	H
ASN	440	R	ARG	105	H
ASN	440	R	LEU	102	H
ASN	440	R	TRP	50	H
ASN	440	R	THR	55	H
ASN	440	R	ASN	52	H
LEU	441	R	ARG	105	H
LEU	441	R	TRP	94	L
LEU	441	R	ASN	52	H
LEU	441	R	TYR	103	H
LEU	441	R	LEU	102	H

LEU	441	R	TRP	50	H
ASP	442	R	LEU	102	H
ASP	442	R	TYR	103	H
SER	443	R	LEU	102	H
SER	443	R	ASN	52	H
SER	443	R	THR	55	H
SER	443	R	TYR	103	H
SER	443	R	ASN	54	H
LYS	444	R	ASN	31	H
LYS	444	R	ASN	54	H
LYS	444	R	LEU	102	H
VAL	445	R	ASN	31	H
VAL	445	R	ASN	54	H
VAL	445	R	ILE	30	H
ASN	448	R	LEU	102	H
ASN	448	R	TYR	103	H
ASN	450	R	TYR	103	H
TYR	451	R	TYR	103	H
PRO	499	R	THR	55	H
PRO	499	R	ASN	54	H
ARG	509	R	TRP	94	L
ARG	509	R	TYR	103	H

Table S3. The list of the binding epitope residues and BD55-3152 Ab residues forming interfacial contacts

RBD Residue	RBD Residue Number	RBD Chain	Antibody residue	Antibody residue number	Antibody chain
ASP	339	R	SER	93	B
ASP	339	R	ALA	92	B
ASP	339	R	THR	94	B
GLU	340	R	ALA	92	B
GLU	340	R	ASP	91	B
GLU	340	R	SER	93	B
GLU	340	R	SER	29	B
VAL	341	R	ALA	92	B
PHE	342	R	GLY	110	A
PHE	342	R	TRP	109	A
ASN	343	R	SER	93	B
ASN	343	R	SER	111	A
ASN	343	R	GLY	110	A
ASN	343	R	THR	94	B
ASN	343	R	ALA	92	B
ASN	343	R	TRP	109	A
ASN	343	R	GLN	30	B
ASN	343	R	TYR	107	A
ALA	344	R	GLY	110	A
ALA	344	R	SER	29	B
ALA	344	R	ALA	92	B
ALA	344	R	SER	93	B
ALA	344	R	TRP	109	A
ALA	344	R	GLN	30	B
THR	345	R	SER	93	B
THR	345	R	GLY	110	A
THR	345	R	TYR	33	B
THR	345	R	TYR	31	B
THR	345	R	SER	111	A
THR	345	R	PRO	112	A
THR	345	R	LEU	113	A
THR	345	R	SER	29	B
THR	345	R	THR	94	B
THR	345	R	GLN	30	B
ARG	346	R	PRO	28	B
ARG	346	R	SER	29	B
ARG	346	R	GLY	67	B
ARG	346	R	TYR	31	B
ARG	346	R	LEU	27	B
ARG	346	R	THR	65	B
ARG	346	R	VAL	32	B

ARG	346	R	GLN	30	B
ARG	346	R	ASP	50	B
PHE	347	R	TRP	109	A
ASN	354	R	PRO	28	B
ASN	354	R	SER	29	B
VAL	367	R	TRP	109	A
LEU	368	R	TRP	109	A
LEU	371	R	TRP	109	A
ALA	372	R	TYR	107	A
PHE	375	R	TRP	109	A
PHE	375	R	TYR	107	A
TRP	436	R	VAL	108	A
TRP	436	R	TRP	109	A
ASN	437	R	VAL	108	A
SER	438	R	VAL	108	A
LYS	440	R	PRO	102	A
LYS	440	R	LEU	103	A
LYS	440	R	PHE	101	A
LYS	440	R	SER	104	A
LYS	440	R	VAL	108	A
LYS	440	R	ASP	105	A
LEU	441	R	TRP	109	A
LEU	441	R	VAL	108	A
LEU	441	R	GLY	110	A
LEU	441	R	PHE	101	A
LEU	441	R	TYR	31	B
LEU	441	R	SER	111	A
LEU	441	R	PRO	112	A
ASP	442	R	TYR	31	B
LYS	444	R	GLU	52	B
ASN	448	R	GLU	52	B
ASN	448	R	TYR	31	B
TYR	451	R	TYR	31	B
ARG	509	R	TRP	109	A
ARG	509	R	GLY	110	A
ARG	509	R	TYR	31	B
ARG	509	R	SER	111	A

Table S4. The list of the binding epitope residues and SA58 Ab residues forming interfacial contacts

RBD Residue	RBD Residue Number	RBD Chain	Antibody residue	Antibody residue number	Antibody chain
PRO	337	R	LEU	29	L
PRO	337	R	SER	28	L
ASP	339	R	ASN	95	L
ASP	339	R	GLU	1	L
GLU	340	R	ALA	27	L
GLU	340	R	SER	28	L
GLU	340	R	ASN	95	L
GLU	340	R	GLU	1	L
GLU	340	R	ARG	26	L
GLU	340	R	GLY	30	L
GLU	340	R	LEU	29	L
GLU	340	R	VAL	2	L
VAL	341	R	ASN	95	L
VAL	341	R	LEU	29	L
ASN	343	R	SER	94	L
ASN	343	R	PRO	97	L
ASN	343	R	ASN	95	L
ASN	343	R	TRP	96	L
ALA	344	R	SER	94	L
ALA	344	R	ASN	95	L
ALA	344	R	TRP	96	L
THR	345	R	TRP	96	L
THR	345	R	TYR	105	H
THR	345	R	ASP	34	L
THR	345	R	TYR	93	L
THR	345	R	LEU	98	L
THR	345	R	SER	94	L
THR	345	R	ASN	95	L
ARG	346	R	PHE	106	H
ARG	346	R	SER	94	L
ARG	346	R	SER	103	H
ARG	346	R	ASP	104	H
ARG	346	R	TYR	105	H
ARG	346	R	ASP	34	L
ARG	346	R	TYR	93	L
LYS	356	R	LEU	29	L
ARG	357	R	LEU	29	L
ILE	358	R	LEU	29	L
LYS	440	R	TRP	50	H
LYS	440	R	THR	57	H
LYS	440	R	ASN	32	H

LYS	440	R	PRO	58	H
LYS	440	R	ASN	52	H
LYS	440	R	TYR	102	H
LYS	440	R	THR	59	H
LEU	441	R	ASN	52	H
LEU	441	R	TRP	96	L
LEU	441	R	TYR	102	H
LEU	441	R	TRP	50	H
LEU	441	R	SER	103	H
LEU	441	R	TYR	105	H
ASP	442	R	TYR	102	H
ASP	442	R	SER	103	H
ASP	442	R	TYR	105	H
SER	443	R	ASP	54	H
SER	443	R	TYR	102	H
SER	443	R	ASN	32	H
LYS	444	R	THR	30	H
LYS	444	R	ASN	32	H
LYS	444	R	SER	31	H
LYS	444	R	ASP	54	H
LYS	444	R	TYR	102	H
VAL	445	R	ASP	54	H
ASN	448	R	TYR	102	H
ASN	448	R	SER	103	H
ASN	450	R	TYR	102	H
ASN	450	R	SER	103	H
TYR	451	R	SER	103	H
ARG	509	R	TYR	105	H
ARG	509	R	TRP	96	L

Table S5. The list of the binding epitope residues and BD55-3372 Ab residues forming interfacial contacts

RBD Residue	RBD Residue Number	RBD Chain	Antibody residue	Antibody residue number	Antibody chain
ALA	372	R	LEU	116	L
ARG	403	R	ASN	50	H
GLY	404	R	SER	75	H
GLY	404	R	PHE	76	H
ASP	405	R	PHE	76	H
ASP	405	R	THR	71	H
ASP	405	R	ASN	50	H
ASP	405	R	SER	73	H
ASP	405	R	SER	75	H
ASP	405	R	GLY	72	H
ASP	405	R	SER	74	H
GLU	406	R	SER	73	H
VAL	407	R	SER	75	H
VAL	407	R	PHE	76	H
ARG	408	R	SER	75	H
ARG	408	R	SER	74	H
ARG	408	R	SER	73	H
GLN	409	R	SER	73	H
ASN	437	R	SER	114	L
ASN	439	R	TYR	52	L
GLN	498	R	ASP	120	H
GLN	498	R	TYR	122	H
GLN	498	R	ASP	121	H
PRO	499	R	TYR	52	L
PRO	499	R	ALA	50	L
PRO	499	R	GLY	51	L
THR	500	R	GLY	51	L
THR	500	R	ASP	123	H
THR	500	R	ASP	121	H
THR	500	R	GLU	53	L
THR	500	R	THR	124	H
THR	500	R	TYR	122	H
THR	500	R	TYR	112	L
THR	500	R	TYR	52	L
ASN	501	R	ARG	119	H
ASN	501	R	SER	114	L
ASN	501	R	TYR	122	H
ASN	501	R	TYR	112	L

ASN	501	R	ASP	120	H
ASN	501	R	TYR	52	L
ASN	501	R	ASP	123	H
ASN	501	R	ASP	121	H
GLY	502	R	TYR	122	H
GLY	502	R	TYR	112	L
GLY	502	R	TYR	52	L
GLY	502	R	ASP	123	H
GLY	502	R	GLU	118	H
VAL	503	R	THR	71	H
VAL	503	R	TYR	112	L
VAL	503	R	SER	119	L
VAL	503	R	TYR	52	L
VAL	503	R	PHE	76	H
VAL	503	R	PHE	78	H
VAL	503	R	SER	114	L
GLY	504	R	PHE	76	H
GLY	504	R	THR	71	H
GLY	504	R	TYR	112	L
TYR	505	R	ASP	120	H
TYR	505	R	ASN	50	H
TYR	505	R	TYR	51	H
TYR	505	R	ASP	123	H
GLN	506	R	TYR	52	L
GLN	506	R	SER	114	L
GLN	506	R	TYR	112	L
TYR	508	R	PHE	76	H

Table S6. The list of the binding epitope residues and BD55-4637 Ab residues forming interfacial contacts

RBD Residue	RBD Residue Number	RBD Chain	Antibody residue	Antibody residue number	Antibody chain
ALA	372	R	LYS	31	A
PRO	373	R	LYS	31	A
PHE	374	R	ASN	32	A
PHE	374	R	LYS	30	A
PHE	374	R	LYS	31	A
PHE	374	R	SER	28	A
PHE	375	R	ASN	32	A
PHE	375	R	LYS	31	A
THR	376	R	ASP	102	A
THR	376	R	LEU	103	A
THR	376	R	LYS	31	A
THR	376	R	LEU	107	A
THR	376	R	ASN	32	A
THR	376	R	GLY	33	A
PHE	377	R	LYS	31	A
PHE	377	R	LEU	107	A
LYS	378	R	SER	105	A
LYS	378	R	LEU	107	A
LYS	378	R	ASP	106	A
ARG	403	R	ARG	31	B
GLY	404	R	VAL	109	A
ASP	405	R	THR	33	B
ASP	405	R	VAL	109	A
ASP	405	R	HIS	51	B
VAL	407	R	ILE	108	A
VAL	407	R	VAL	109	A
VAL	407	R	LEU	107	A
ARG	408	R	ILE	108	A
ARG	408	R	LEU	107	A
ARG	408	R	ASP	106	A
GLN	414	R	ASP	106	A
VAL	433	R	LEU	107	A
ALA	435	R	ASN	32	A
ALA	435	R	LEU	107	A
TRP	436	R	ASN	32	A
ASN	437	R	ASN	32	A
ASN	437	R	GLY	33	A
ASN	437	R	ASP	102	A
ASN	437	R	TRP	55	A
ASN	439	R	TYR	54	A

ASN	439	R	SER	58	A
ASN	439	R	ASP	56	A
ASN	439	R	ARG	60	A
LYS	440	R	ASP	56	A
LYS	440	R	SER	58	A
SER	496	R	ARG	31	B
ARG	498	R	ARG	31	B
ARG	498	R	ASP	94	B
PRO	499	R	ARG	60	A
THR	500	R	ASP	93	B
THR	500	R	SER	97	B
THR	500	R	SER	95	B
THR	500	R	ARG	60	A
THR	500	R	TRP	92	B
THR	500	R	ASP	94	B
TYR	501	R	TRP	92	B
TYR	501	R	ARG	31	B
TYR	501	R	ARG	60	A
TYR	501	R	ASN	32	B
TYR	501	R	ASP	94	B
GLY	502	R	ASN	32	B
GLY	502	R	TRP	92	B
GLY	502	R	ASP	94	B
GLY	502	R	ASP	111	A
VAL	503	R	ASP	102	A
VAL	503	R	VAL	109	A
VAL	503	R	TRP	92	B
VAL	503	R	ASP	111	A
VAL	503	R	PRO	101	A
VAL	503	R	ASN	32	B
VAL	503	R	TYR	54	A
GLY	504	R	ASN	32	B
GLY	504	R	VAL	109	A
GLY	504	R	ASP	111	A
HIS	505	R	ASP	94	B
HIS	505	R	ARG	31	B
HIS	505	R	ASN	32	B
GLN	506	R	ARG	60	A
GLN	506	R	TYR	54	A
GLN	506	R	TRP	55	A
TYR	508	R	GLY	33	A

Table S7. The list of the binding epitope residues and SA55 Ab residues forming interfacial contacts

RBD Residue	RBD Residue Number	RBD Chain	Antibody residue	Antibody residue number	Antibody chain
PRO	373	R	LEU	94	B
PHE	374	R	THR	57	A
PHE	374	R	PHE	55	A
THR	376	R	PHE	55	A
ARG	403	R	PRO	105	A
ARG	403	R	ASN	106	A
GLY	404	R	PHE	55	A
GLY	404	R	LEU	54	A
GLY	404	R	ARG	30	A
ASP	405	R	LEU	54	A
ASP	405	R	SER	31	A
ASP	405	R	THR	28	A
ASP	405	R	ARG	30	A
GLU	406	R	ARG	30	A
VAL	407	R	ARG	30	A
VAL	407	R	PHE	55	A
VAL	407	R	LEU	54	A
ARG	408	R	ARG	30	A
ASN	437	R	ASP	93	B
ASN	439	R	TYR	91	B
ASN	439	R	ASP	93	B
LYS	440	R	ASP	93	B
VAL	445	R	HIS	53	B
TYR	495	R	PRO	105	A
SER	496	R	PRO	105	A
ARG	498	R	PHE	112	A
ARG	498	R	TYR	49	B
PRO	499	R	PHE	100	A
PRO	499	R	PRO	101	A
PRO	499	R	ASP	50	B
PRO	499	R	TYR	91	B
THR	500	R	GLY	103	A
THR	500	R	PHE	112	A
THR	500	R	PHE	100	A
THR	500	R	TYR	49	B
THR	500	R	ASP	104	A
THR	500	R	PRO	101	A
THR	500	R	ASN	102	A
THR	500	R	ASP	50	B
TYR	501	R	PRO	105	A

TYR	501	R	GLY	103	A
TYR	501	R	PHE	112	A
TYR	501	R	ASN	102	A
TYR	501	R	ASP	104	A
TYR	501	R	PRO	101	A
GLY	502	R	ASP	104	A
GLY	502	R	PRO	101	A
GLY	502	R	SER	31	A
GLY	502	R	GLY	103	A
GLY	502	R	HIS	32	A
GLY	502	R	ASN	102	A
VAL	503	R	PRO	95	B
VAL	503	R	PHE	55	A
VAL	503	R	VAL	33	A
VAL	503	R	ASN	102	A
VAL	503	R	LEU	54	A
VAL	503	R	HIS	32	A
VAL	503	R	PRO	101	A
VAL	503	R	ILE	52	A
VAL	503	R	SER	31	A
GLY	504	R	LEU	54	A
GLY	504	R	HIS	32	A
GLY	504	R	SER	31	A
GLY	504	R	ARG	30	A
HIS	505	R	PRO	105	A
HIS	505	R	HIS	32	A
HIS	505	R	ASP	104	A
HIS	505	R	SER	31	A
HIS	505	R	GLY	103	A
GLN	506	R	PRO	101	A
GLN	506	R	TYR	91	B
GLN	506	R	ASP	93	B
TYR	508	R	LEU	54	A
TYR	508	R	PHE	55	A
